# Supplementary material for: TopNEXt: automatic DDA exclusion framework for multi-sample mass spectrometry experiments
Source: Bioinformatics. 2023 Jun 26;39(7):btad406. doi: 10.1093/bioinformatics/btad406 (PMC10336026; doi:10.1093/bioinformatics/btad406)
Supplement: btad406_Supplementary_Data [file btad406_supplementary_data.pdf]

TopNEXt: Automatic DDA Exclusion Framework for Multi-Sample Mass Spectrometry Experiments – Supplementary Information

Ross McBride[1, *, †], Joe Wandy[2, †], Stefan Weidt[2], Simon Rogers[1], Vinny Davies[3, *, ‡], Rónán Daly[2, ‡], and Kevin Bryson[1, ‡]

[*]To whom correspondence should be addressed.
[†] The authors wish it to be known that, in their opinion, the first two authors should be regarded as Joint First Authors.
[‡] The authors wish it to be known that, in their opinion, the last three authors should be regarded as Joint Last Authors.

[1]School of Computing Science, University of Glasgow, Glasgow, United Kingdom.
[2]Glasgow Polyomics, University of Glasgow, Glasgow, United Kingdom.
[3]School of Mathematics and Statistics, University of Glasgow, Glasgow, United Kingdom.

1 Beer Data

For our experiments we collected samples of ten arbitrarily-chosen store-bought beers. Store-bought beers are metabolomically complex enough to be a useful test of fragmentation strategy performance, but are also straightforward to obtain and use (alternatives might introduce ethics or bio-safety concerns) which eases reproduction of our results. The list can be found in Table 1. Note that only the first six beers were used for our experiments in the main paper: all ten were used for a simulated replication study which can be found later in this supplementary information.

Index	Name	Type
1	Raspberry Sour by Vault City	Sour
2	Cacao & Hazelnut Broken Dream Twisted Breakfast Stout by Siren	Stout
3	Tennents	Lager
4	Life and Death by Vocation	IPA
5	Silence is... Citra by Overtone	Pale Ale
6	Punk AF by Brewdog	Alcohol-Free IPA
7	The Hop - Single Hop Series Simcoe Edition by Salt	IPA
8	42 DDH Pale Ale by BBNO	Pale Ale
9	Aoraki by Vocation	DIPA
10	Punk IPA by Brewdog	IPA

Table 1: A complete list of the beers collected for our experiments. Note that although the specific choices of sample were arbitrary, we varied the class of beer in the “Type” column in an attempt to obtain varied metabolic profiles.

For the replications of the 6-4 experiment we had each observation randomly sample six beers out of the ten. The random samples we used are in Table 2.

Observation Num.	Beer Indices
1	(2, 1, 8, 4, 5, 10)
2	(9, 4, 5, 7, 10, 1)
3	(3, 8, 9, 5, 1, 2)
4	(9, 4, 2, 7, 8, 5)
5	(5, 6, 4, 7, 9, 2)
6	(7, 4, 8, 9, 10, 5)
7	(8, 5, 10, 2, 9, 3)
8	(7, 4, 5, 10, 1, 2)
9	(8, 6, 10, 3, 7, 2)
10	(7, 1, 2, 10, 9, 3)

Table 2: The random samples used for the 10 replications in the 6-4 replicate experiments.

2 Peak-Picking Parameters

2.1 Peak-Picking

To compute our evaluation metrics, we used MZMine 2 (version 2.53) [3] with a fullscan .mzML as the input, producing as output a set of peak-boxes indicating regions we would ideally like to fragment for that sample. For a re-simulated experiment, we used the same fullscan for generation of re-simulations and for creation of peak-boxes. After using the ADAP [2] chromatogram builder and deconvolver and grouping isotopes, we aligned all samples in each experiment. For example, the six different sets of peak-boxes produced by the six different samples in the 6-4 beers experiment. This produces a matrix where rows give a single aligned peak, and columns the sample the peaks were observed in. For each fragmentation run our ViMMS code updates its corresponding column with information like maximum intensities and fragmentation intensities — this allows computing coverage and intensity coverage by collapsing rows and columns.

To produce our steps from chromatogram detection to alignment, we defined an MZMine .xml template file which specifies the order of steps and their parameters (these "batch mode" files can be created and edited through the MZMine GUI). To run this from our codebase we insert the names of the input/output files into a base template .xml, and call MZMine from the command line using Python's subprocess module and this modified template. We have two base templates — "a permissive" parameter set previously used to evaluate SmartRoI and WeightedDEW [1] and a "restrictive" parameter set which was further refined by our mass spectrometry expert. Both of these are available on the ViMMS GitHub repository. The restrictive parameter set filters out lower-quality peaks and more closely represents actual practise, therefore we primarily used this set (including for everything in the main paper) when evaluating our results. The permissive parameter set produces a larger set of peak-boxes and therefore may give us some insight into behaviour on samples more dense with peaks, so we have used it for some additional experiments in this supplementary document. The complete list of batch steps and parameter settings can be found in Tables 3 and 4 — italics are used in the restrictive parameter table to highlight changes in parameter values from the permissive table.

Note that there is one change between our permissive parameter set and the set used for the SmartRoI publication [1]. MZMine requires both a Dalton and ppm value to be specified for m/z tolerances, and will use the maximum of these two values. In order to ensure the ppm value will always be used, we set a dummy value of $10^{(-8)}$ for the absolute tolerance.

MZMine Parameters (Permissive)	
**Raw Data Import**	
**Crop Filter**	
Retention time	0.5 to 30 minutes
m/z	50 to 1060 Da
**Mass Detection (MS1)**	
MS Level	1
Mass Detector	Centroid
Noise Level	1000
**Mass Detection (MS2)**	
MS Level	2
Mass Detector	Centroid
Noise Level	0
**ADAP Chromatogram Builder**	
MS Level	1
Min group size in # of scans	3
Group intensity threshold	500
Min highest intensity	5000
m/z tolerance	$10^{(-8)}$ Da or 10.0 ppm
**Chromatogram Deconvolution**	
Algorithm	Wavelets (ADAP)
S/N Threshold	10
S/N Estimator	Intensity window SN
Min feature height	5000
Coefficient/area threshold	1
Peak duration range	0.10 to 5.00
rt wavelet range	0.10 to 1.00
m/z centre calculation	Median
m/z range for MS2 scan pairing (Da)	Enabled, 0.01
rt range for MS2 scan pairing (minutes)	Enabled, 0.5
**Isotopic Peaks Grouper**	
m/z tolerance	$10^{(-8)}$ Da or 10.0 ppm
Retention time tolerance	0.1, absolute (minutes)
Monotonic shape	False
Maximum charge	2
Representative isotope	Most intense
**Join Aligner**	
m/z tolerance	$10^{(-8)}$ Da or 10.0 ppm
Weight for m/z	80
Retention time tolerance	0.1, absolute (minutes)
Weight for rt	30
Require same charge state	False
Compare isotope pattern	False
Compare spectra similarity	False
**Export to CSV**	

Table 3: A table of “permissive” peak-picking parameters used in previous work [1] and for our additional experiments.

MZMine Parameters (Restrictive)	
**Raw Data Import**	
**Crop Filter**	
Retention time	0.5 to 30 minutes
m/z	50 to 1060 Da
**Mass Detection (MS1)**	
MS Level	1
Mass Detector	Centroid
Noise Level	1000
**Mass Detection (MS2)**	
MS Level	2
Mass Detector	Centroid
Noise Level	0
**ADAP Chromatogram Builder**	
MS Level	1
Min group size in # of scans	3
Group intensity threshold	*2000*
Min highest intensity	5000
m/z tolerance	*$10^{(-8)}$ Da or 5.0 ppm*
**Chromatogram Deconvolution**	
Algorithm	Wavelets (ADAP)
S/N Threshold	*3*
S/N Estimator	Intensity window SN
Min feature height	5000
Coefficient/area threshold	1
Peak duration range	*1.0 to 7.00*
rt wavelet range	*1.00 to 5.00*
m/z centre calculation	Median
m/z range for MS2 scan pairing (Da)	Enabled, 0.01
rt range for MS2 scan pairing (minutes)	Enabled, 0.5
**Isotopic Peaks Grouper**	
m/z tolerance	*$10^{(-8)}$ Da or 5.0 ppm*
Retention time tolerance	0.1, absolute (minutes)
Monotonic shape	False
Maximum charge	2
Representative isotope	*Lowest m/z*
**Join Aligner**	
m/z tolerance	*$10^{(-8)}$ Da or 5.0 ppm*
Weight for m/z	80
Retention time tolerance	*0.5, absolute (minutes)*
Weight for rt	20
Require same charge state	False
Compare isotope pattern	False
Compare spectra similarity	False
**Export to CSV**	

Table 4: A table of more realistic “restrictive” parameters used for most of our fragmentation strategy evaluations, including those in the main paper.

3 Metrics

As we elaborated on in the Methodology section of the main manuscript, *peak coverage* (i.e. coverage) and *intensity coverage* are our two measures of DDA strategy performance. Again, given some set of detected

peaks (in our case, from MZMine peak-picking) coverage measures how many detected peaks we have with at least one fragmentation spectra above a minimum intensity threshold. For each peak-box, we award a point to a fragmentation run output for each peak-box which has both a fragmentation event and a valid precursor for that fragmentation event. For each fragmentation event we create a one-dimensional m/z interval (i.e. isolation window) of a user-specified length around its centre, and any peak-box that is completely covered by it on the m/z dimension is considered to be fragmented by it. Any point from the previous MS1 scan above the minimum intensity threshold and within the peak-box is then a precursor to this fragmentation event. The *coverage* is then just the count of peak-boxes for which these two conditions are met. The *cumulative coverage* is the same count with aligned peak-boxes across a multi-injection experiment — each peak is aligned one point if it is fragmented in *any* sample and no more than one for being fragmented across several. For each sample we used the original location of that peak-box in the individual sample, not the aligned summary box. Our results present the (cumulative) coverage as a $[0, 1]$-bounded proportion of the total possible (cumulative) coverage.

Intensity coverage generalises this idea by measuring the aggregate quality of these acquisitions. This is done by replacing the binary $0, 1$ score awarded to each peak with a proportion of the maximum fragmentation intensity to the maximum *possible* fragmentation intensity i.e. a score in a $[0, 1]$ range. The maximum fragmentation intensity is just the maximum intensity of any of the fragmentation event's precursors, or 0 if there are none above the minimum threshold; the maximum possible fragmentation intensity is just the maximum intensity of the peak i.e. the maximum intensity of any MS1 points falling within its peak-box in one of the fragmentation runs. We then compute *intensity coverage* and *cumulative intensity coverage* in a similar way to coverage. However, note that when computing maximum intensities for aligned peak-boxes, we use their locations in individual samples but their maximum intensity across all samples.

Finally, note that for all evaluations of a given sequence of fragmentation runs we only include peaks which had a maximum intensity above a minimum threshold in one of those fragmentation runs. This can cause slight inconsistencies between experiment cases (especially if one of them schedules more MS1 scans) but avoids penalising fragmentation strategies for peaks that are completely unobtainable. Furthermore, note that these "window mode" measurements of the coverage and intensity coverage are different from previous work, which used "point mode" [1]. In point mode we treat each fragmentation event as a point rather than an isolation window, and check if the point falls within the peak-box, and assign precursors if they fall within an error tolerance (e.g. 10 ppm).

Coverage and intensity coverage give us a direct measure of how well we are addressing DDA's typical weaknesses. Additionally, we avoid any real-world complications which are not directly relevant to the question we are asking — for example, to meaningfully evaluate any data by metabolite annotation, we would have to have a (relatively) complete and accurate database of metabolites for the sample, whereas evaluating by coverage does not depend on our current knowledge, provided our peak-boxes highlight every region of interest. Even if we do not know which of these peaks correspond to metabolites, if we collect strictly more of them at better qualities, it follows that we will have more correct metabolite identifications, and fewer misidentifications. However, while all our methods presented here build on TopN, so it is unlikely the distribution of peaks they target differs significantly, this remains a potential difficulty. And with intensity coverage, would it be better to collect one peak at 25% of maximum and another at 75%, or to collect two at 50%? Improvements on low-intensity peaks are likely to be more valuable, but intensity coverage will score them equally as long as they are above the fragmentation threshold. Additionally, these metrics cannot make a meaningful comparison to DIA, which can cover all peaks but must then recover usable information from them. Future work will have to compare DDA to pre-scheduling and DIA, but coverage will still be useful in disentangling where differences in metabolite annotations come from.

4 Controller Parameters

In order to ensure that the parameters used for dynamic exclusion had reasonable values, we grid-searched a small range of plausible values. These and shared parameter values can be found in the tables below. Highlighted in bold are the values found to be optimal when searching and therefore used for our experiments. The parameters of the actual WeightedDEW implementation slightly differ from the presentation in Section 6.1: rt_tol expresses the total rt-length of window d_0 plus d_1. We list the values actually used in the code

here. Invalid combinations caused by rt_tol having a strictly lesser value than exclusion_t_0 (i.e. d_0) were excluded. mz_tol indicates the ppm mass tolerance used for RoI-building/the fixed size of exclusion windows in the case of TopN Exclusion. min_roi_length indicates how many points an RoI must have at minimum before it is discarded.

**Shared Parameters**	
Ionisation mode	Positive
Isolation width	1
Min MS1 intensity (λ_{min})	5000
mz_tol	10
min_roi_length	3

Table 5: Shared controller parameters that were used throughout all our parameter optimisations and experiments. For the real experiments we instead used an isolation width of 0.7 — this is the minimum our instrument supports.

**Grid Search Parameter Values**	
**Standard DEW**	
N	1, 3, 5, 10, **20**
rt_tol	15, 30, **60**, 120, 240
**SmartRoI**	
N	1, 3, 5, 10, **20**
rt_tol	**15**
intensity_increase_factor (α)	2, **3**, 5, 10
drop_perc (β)	0, $\mathbf{10^{-3}}$, 10^{-2}, 10^{-1}
**WeightedDEW**	
N	1, 3, 5, 10, **20**
exclusion_t_0 (d_0)	**1**, 10, 15, 30, 60
rt_tol ($d_0 + d_1$)	15, 30, **60**, 120, 240

Table 6: Searched parameter values for each DEW variant. All combinations for a given variant were tried: marked in bold are the highest-scoring values, which we used for our actual experiments.

In total there were 227 searched cases. In each case we had ViMMS generate a set of chemical objects from the real fullscan .mzMLs generated for beers 1, 2 and 3. Then for each parameter combination, we ran a smaller version of the 6/4 experiment with 6 total runs following the order 1-2-3-1-2-3. All of these parameter combinations were then sorted by their proportional intensity coverage, and the parameter values used in our actual experiments were the ones with the highest proportional intensity coverage. The fullscans used were generated as part of our lab experiment on the first day, and thereafter we used the parameter values optimised on these. Additional fullscans were generated for use with the fragmentation runs on other days, but the length of the optimisation procedure prevented these being used to optimise parameters for the data generated on the same day.

Although this procedure significantly improved the results across all controllers when comparing re-simulated results (mainly due to increasing the value of the "N" parameter from its initial value of 10) this is not an exhaustive parameter search. As this procedure can be quite time-intensive, the individual domains for the parameters we considered were relatively small, and the 3-2 experiment might behave differently from a 6-4 or 1-20 setup. We found that when ranking these parameter combinations by intensity coverage, WeightedDEW combinations appeared before regular DEW which appeared before SmartRoI. This result was consistent when optimising parameters both on preliminary data and the fullscans generated on day 1 of our lab experiment, so we used WeightedDEW in the lab experiment. However, in our final results,

SmartRoI consistently performed the best. We speculate that the different sample setup (3-2 vs 6-4 or 1-20) is the reason for this. Exhaustive comparisons are left to future work.

Additionally, all the controllers share the same dynamic exclusion parameters, so we conducted this search using the Intensity Non-Overlap controller, under the reasoning that it would be best to search using the controller with the most complicated behaviour. However, we could suppose the most dissimilar controller from Intensity Non-Overlap in our comparison, TopN Exclusion, interacts significantly differently with different parameter values. Therefore as a basic sanity check we re-optimised the parameters for the regular DEW using TopN Exclusion instead, and compared TopN Exclusion to Intensity Non-Overlap in a re-simulated experiment. The optimal value of $N = 20$ did not change, but TopN Exclusion instead had a slight preference for $rt\_tol = 30$ rather than a value of 60, so we used this value for both controllers.

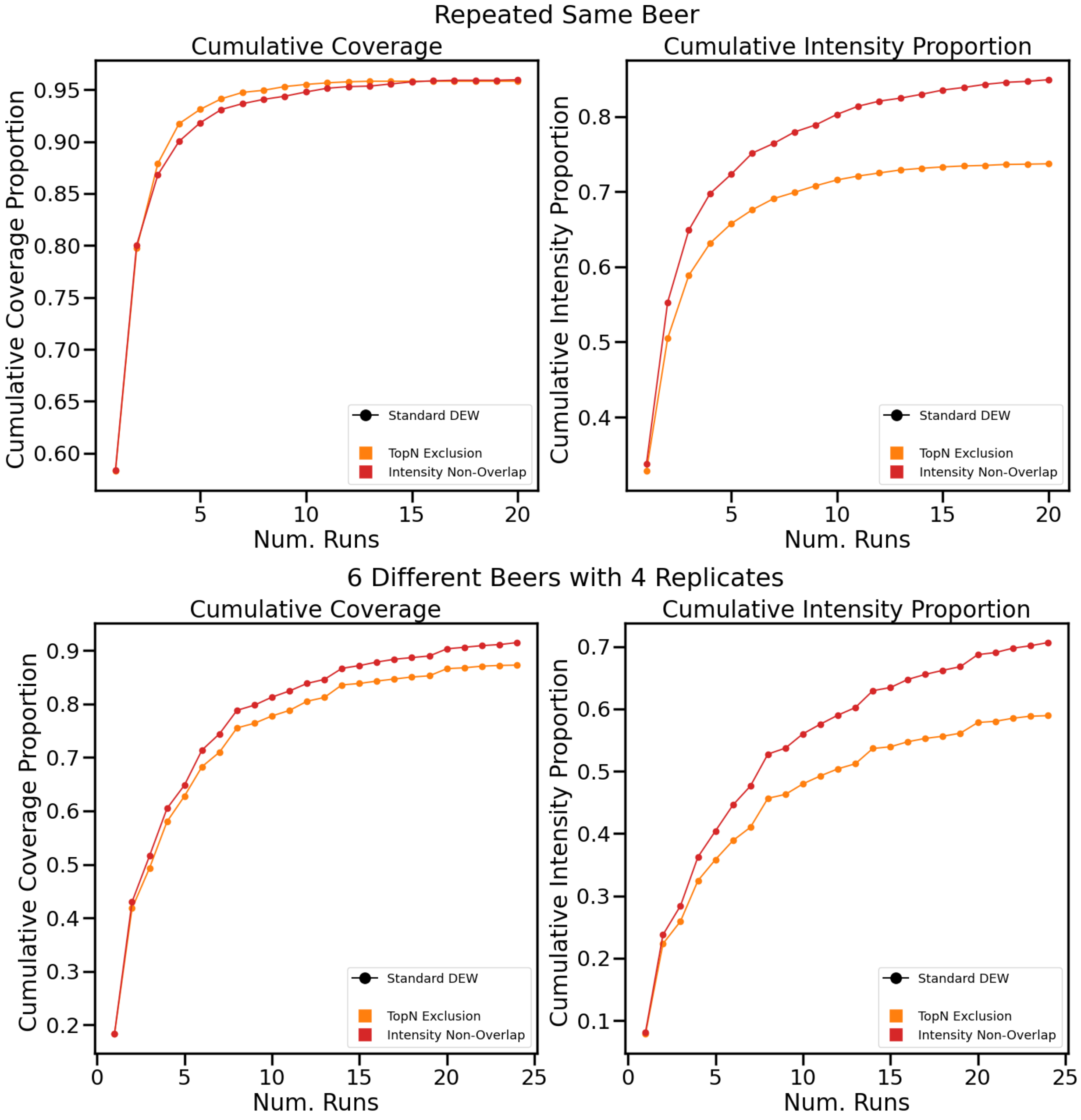

Figure 1: Comparison between TopN Exclusion and regular DEW Intensity Non-Overlap using optimal values for TopN Exclusion and restrictive peak-picking. Top: same beer repeated. Bottom: 6-4 beers.

We can observe in Fig. 1 that this does not significantly change the results of the evaluation. The methods are still competitive in terms of repeated sample coverage, Intensity Non-Overlap has a slight advantage in coverage in the multi-sample case, and Intensity Non-Overlap still has a strong advantage in intensity coverage in both cases.

5 Extended Results

All results in the main paper use the same set of real beer data, either from the lab experiment or re-simulated from some of the fullscans produced by it. These were then all evaluated using the restrictive parameter set elaborated on in 2. To give a better idea of the behaviour when generalised to other scenarios, this section contains several alternative experiments.

5.1 Replication Study

It could be argued that the experiments in the main paper may not be reproducible on even our other beer samples, due to only being repeated once. To address this concern we have performed a simulated replication study. Here we repeated both experiments ten times, and aggregated the results per controller — for the same beer experiment we have used a different beer for each replication, and for the 6-4 experiment we have randomly sampled a different set of six beers out of the total set of ten and had each controller perform the 6-4 experiment with these ten sets of six beers. The beers chosen for the 6-4 experiment can be found in Table 2.

In Figures 2 and 3 we see the final coverage and intensity coverage for each of these sets of repeated experiments. The results are highly similar to the individual experiments we have seen in the main paper. For the same beer case, all methods except TopN finished with near-total coverage, and the intensity methods perform best on intensity coverage, with Intensity Non-Overlap having a spike of around 10% compared to non-intensity methods. In the 6-4 case, we again observe that the new methods are a significant improvement on coverage, with Intensity Non-Overlap having a marginal advantage over the others. And for intensity coverage we see again that the new methods performed better, with the Intensity Non-Overlap having the largest advantage. As before, the difference between Intensity Non-Overlap and the TopN Exclusion variants in this case is almost a fifth of the total intensity coverage that can possibly be obtained. In total running these experiments produced $1800 + 4320 = 6120$ .mzMLs across forty-seven hours, using 20 computer cores. For comparison, if we were to run these experiments on our mass spectrometer at 26 minutes per .mzML produced, assuming no downtime between runs, then these experiments would take a total of 110.5 mass spectrometer days. Seeing that these results are consistent under all of these conditions we can therefore be confident that they hold across our selection of beers.

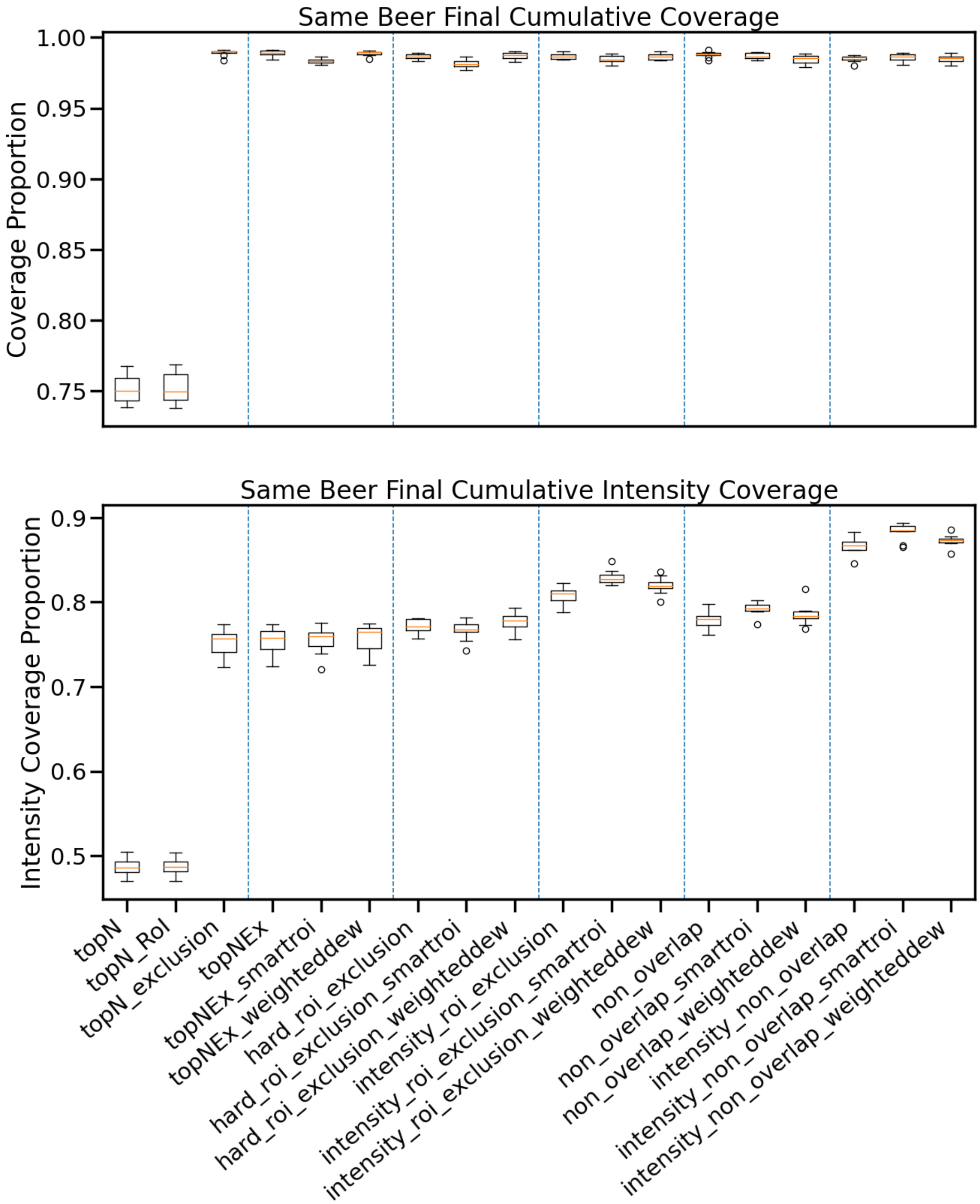

Figure 2: Replicated experiment where for each box there are ten observations, and each observation takes one out of a set of ten different beers (such that all ten beers are each used once per box) and performs a simulated MS experiment with ten repeats of that single beer.

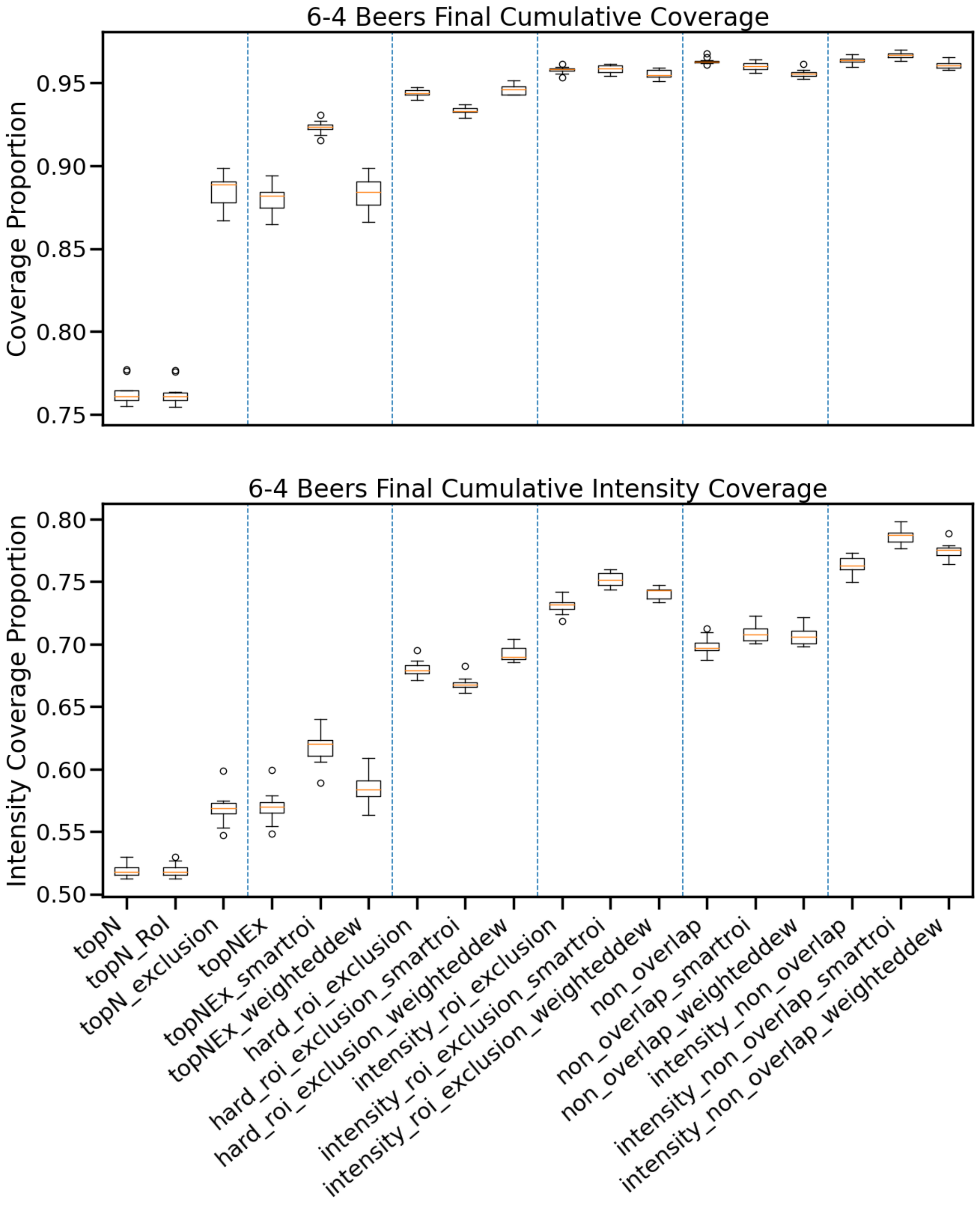

Figure 3: Replicated experiment where for each box there are ten observations and each observation uses a set of six beers that were chosen beforehand by randomly sampling six of the beers, such that each box uses the same set of ten sets of six beers. These six beers are then each injected four times in a simulated experiment to form the single observation.

5.2 Results with Alternative Peak-Picking

Now we ask what happens if we evaluate the lab results using the permissive peak-picking parameter set described in Section 2 rather than the restrictive set we have used up to this point. The values of the restrictive set were chosen in discussion with our mass spectrometry expert and by design filter out a lot of noise, though they will still likely produce significantly more peaks than there are actual annotatable metabolites. Using the permissive parameter set defines a much greater number of peaks as interesting. This creates a much denser and harder problem, but some of those additional "interesting" peaks may represent a low-intensity metabolite. And, if we could get 100% coverage on the permissive parameter set, we would have done so on the restrictive set as well. Therefore we treat this as a proxy for the behaviour of our controllers on samples that even with a restrictive parameter set still end up dense with interesting peaks.

To give an idea of the actual numbers of peaks involved, restrictive peak-picking on the first beer only produced 2148 identified peaks, but permissive parameters produced 10939. Similarly, for the six beers used in the main 6-4 experiment, restrictive peak-picking parameters produced 6490 identified aligned peaks, and permissive parameters produced 22516.

Figure 4 (top) shows the result of the lab experiment with the same beer repeated 10 times from the main paper, but evaluated using the permissive set of MZMine parameters. This time TopN Exclusion has a noticeable coverage advantage of around 5% over Intensity Non-Overlap, and the intensity coverage disadvantage has shrunk to around 8%. A similar change can be observed with the 6-4 beers evaluated using the permissive parameters in Figure 4 (bottom), where TopN Exclusion has a coverage lead of up to around 3% throughout most of the experiment, but is eventually barely overtaken by the overlap methods near the end of the experiment. Intensity Non-Overlap finishes with only around 7% more intensity coverage. Additionally, we can see that (as we would expect) scores for all methods are lower compared to the restrictive parameter set. For the same beer experiment with the restrictive parameters, all methods quickly obtained comprehensive coverage, with TopN Exclusion effectively no longer increasing in coverage by the 8th sample. With the permissive set, TopN Exclusion (the method with the highest coverage) is only slightly above 80% coverage. With the restrictive set, Intensity Non-Overlap had around 86% intensity coverage, but here has only approximately 63%. A similar result can be seen for the 6-4 beers. TopN has only approximately 49% coverage compared to the approximately 77% it was able to obtain with the restrictive parameters, and has only roughly 35% intensity coverage compared to the restrictive 53%. Similarly the strongest methods only break 70% coverage and 60% intensity coverage compared to the thresholds of 90% and 70% seen with the restrictive parameters.

It is clear from the results in Figure 4 that the permissive parameter set makes the coverage problem significantly harder, and that it gives TopN Exclusion a coverage advantage compared to the new methods. (The performance of the new methods relative to each other has not changed much.) The intensity coverage advantage of the new methods shrinks as well, but it is important to note that intensity coverage is not independent of coverage: if a peak is not covered, then it counts as having an intensity coverage of 0%. In Figure5 we see the intensity coverage of these experiments given that we only include peaks which we have covered for that method in the calculation. That is, this is the average intensity proportion where the denominator uses only peaks we have collected so far rather than the entire dataset of peaks. For the same beer case, we see that the difference in this form of intensity coverage between Intensity Non-Overlap and TopN Exclusion is around 13%, but in the 6-4 case it is only approximately 10%. We can also see that in the 6-4 experiment TopN scores better than anything other than the intensity methods on this alternative intensity coverage measure. This measure simply ignores those peaks which TopN did not cover, which is more than half of the total number. Consequently, the fact that TopN has neglected those peaks to target more fragmentation events at those it did cover benefits TopN on this measure. As we have seen previously [1] having a lot of fragmentation events targeted at a single peak increases the chance that one will fall at the optimal time for fragmentation — whether these fragmentation events target similar locations within-sample or between-samples.

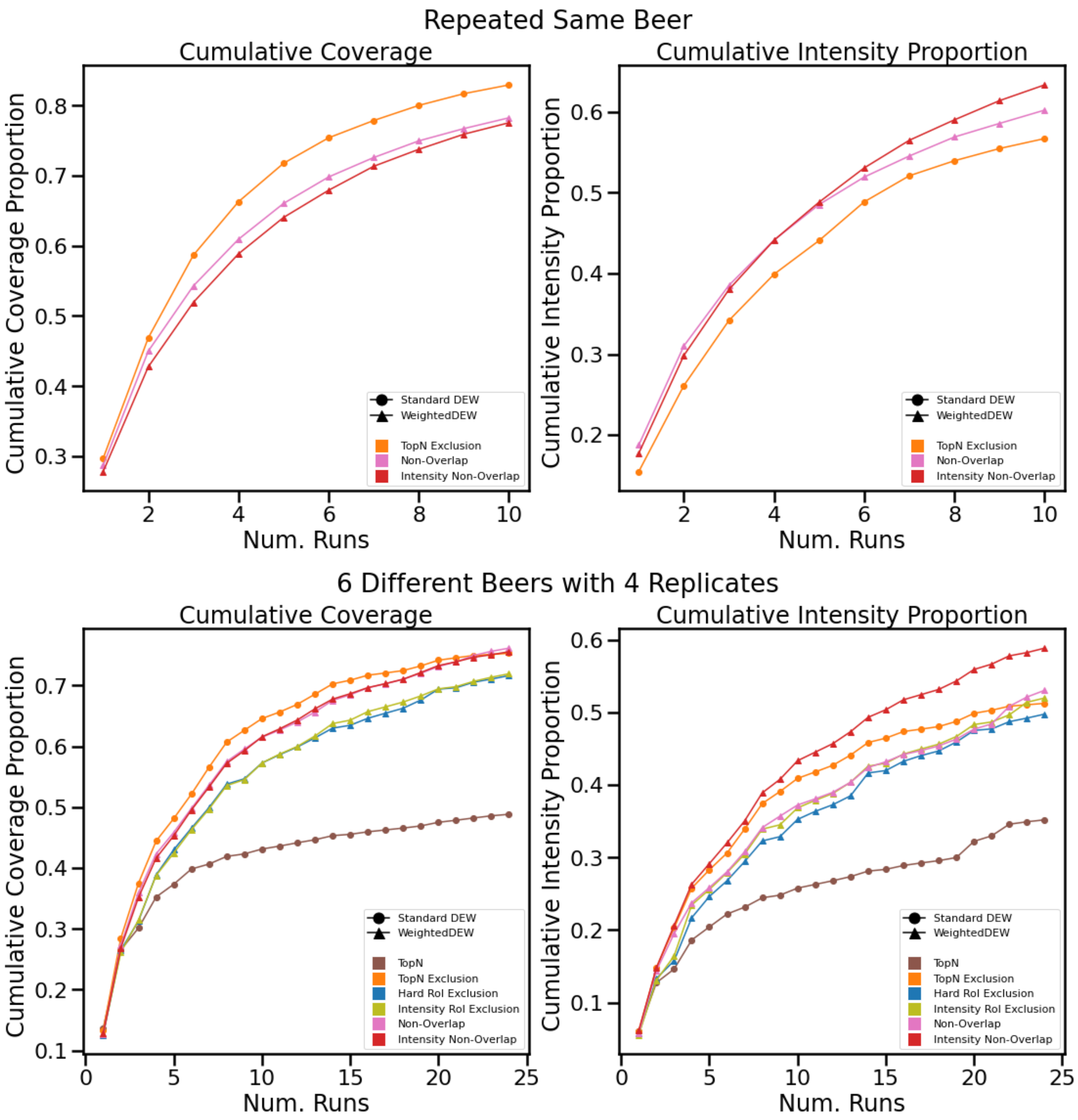

Figure 4: Real experiments evaluated using the permissive parameter set. Top: same beer repeated. Bottom: six different beers each repeated four times.

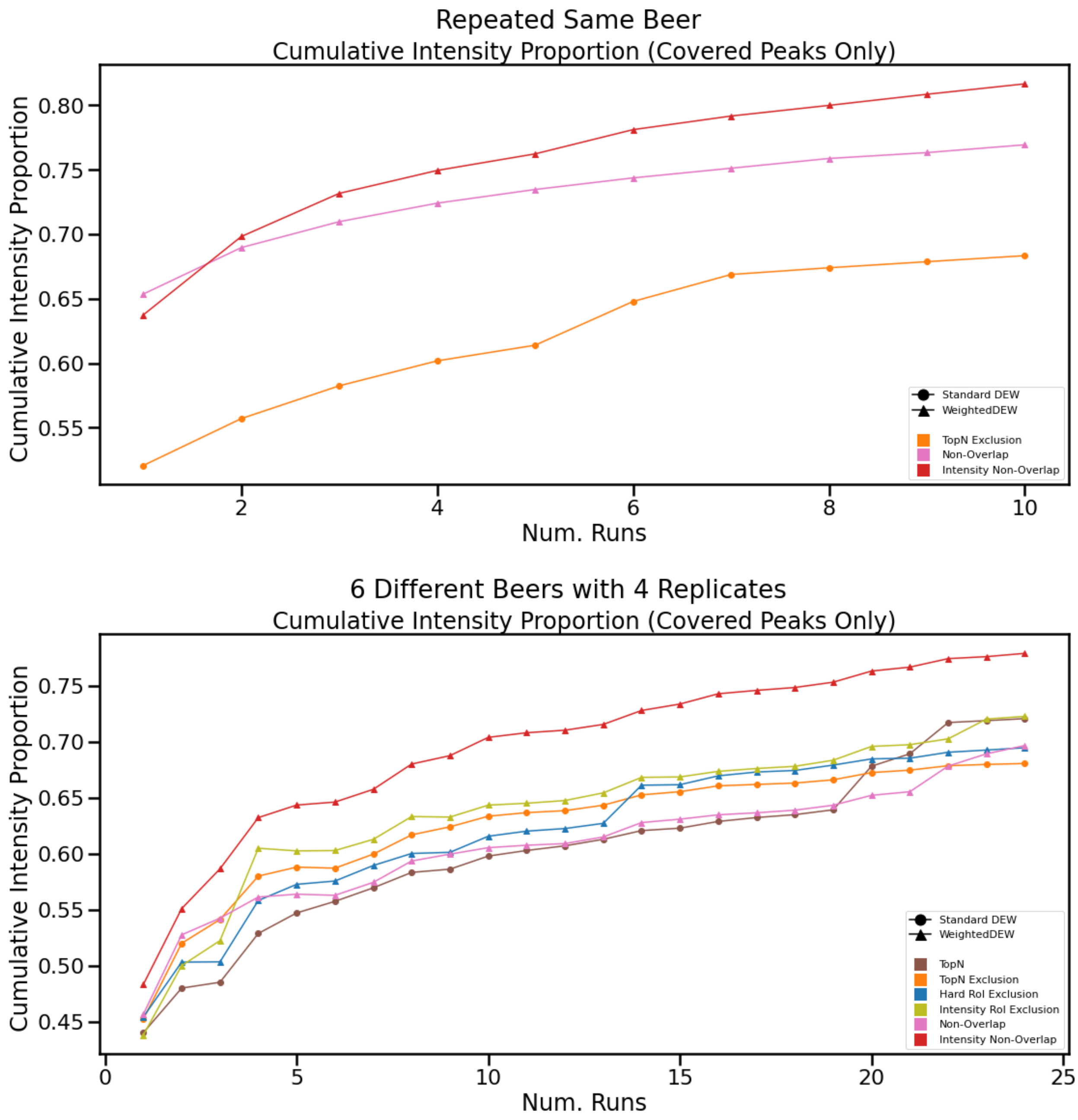

Figure 5: Real experiment evaluated using the permissive parameter set, but showing the intensity proportion where we only include those peaks we have covered. Top: same beer repeated. Bottom: six different beers each repeated four times.

However, even though the relative coverage of Intensity Non-Overlap compared to TopN Exclusion improves from the same beer experiment to the 6-4 beers, the gap in quality of the spectra they do have narrows from 13% to 10%. That is, the spectra we do have with Intensity Non-Overlap are significantly higher quality compared to TopN Exclusion, and this difference is *larger* in the case where the coverage is worse. That most likely indicates that the fragmentation events used to broaden TopN Exclusion's coverage on "weak" peaks not included in the restrictive parameter set are instead being used by the overlap methods to strengthen intensity coverage on the "strong" peaks, even beyond the expected fact that Intensity Non-Overlap attempts to specifically target this behaviour.

Therefore one possible explanation for the results we have seen is that our new methods are more likely to revisit regions of the space compared to TopN Exclusion. By revisiting "strong" peaks instead of visiting "weak" peaks or stopping the duty cycle early and scheduling another MS1 scan, we increase the chance that get a higher intensity coverage on those "strong" peaks or obtain similar peaks across multiple samples. If relatively "strong" peaks are the only things included in the evaluation (as with the restrictive parameter set) then naturally this confers an advantage.

To further substantiate the hypothesis that the new methods revisit potential peaks more often, Tables 7 and 8 show counts of fragmentation events per controller. As the number of runs increases, the number of valid targets for fragmentation decreases and so MS1 scans are scheduled instead of MS2 scans. In the simulated experiments we can see that over the course of the experiment TopN Exclusion eventually almost entirely stops scheduling new fragmentation events, while Non-Overlap decreases significantly and Intensity Non-Overlap remains nearly constant. The likely implication of this is that TopN Exclusion is running out of targets because it does not revisit them, whereas Non-Overlap and Intensity Non-Overlap do and thus continue to have targets to schedule fragmentation events on. One further piece of evidence is shown in Figures 6 and7 which show counts of the number of injections each peak was covered in for both experiments with both peak-picking parameter sets. In each case it can be seen that coverage is distributed more heavily towards repeated fragmentations for Non-Overlap and especially Intensity Non-Overlap. This confirms that these methods are more likely to revisit previously fragmented peaks, and together with the fragmentation counts suggests that TopN Exclusion neglects doing this in favour of scheduling MS1 scans.

Another possible explanation is that because TopN Exclusion more often schedules MS1 scans instead of MS2, then it is more likely to see opportunities where "borderline" peaks are the most appealing option. Also, while the evaluation for an individual controller does not include any peaks that do not have a single eligible precursor above the intensity threshold in one of the fragmentation runs tied to that controller, it is also still possible that the permissive parameter set allows many artifacts or otherwise unreachable peaks. Additional MS1 scans may allow these to be considered for fragmentation by the fragmentation strategy.

With both of these behaviours in mind, consider Tables 9 and 10. Table 9 divides the intensity range into powers of 10 (with a minimum cutoff at 5000, the minimum fragmentation intensity) and then shows counts for the numbers of peaks that fell into each bin based on their maximum observed intensity (not fragmentation intensity) during the fragmentation run. It also gives means and medians of the number of injections each peak was covered in, again separated into these bins. Table 10 shows the same information, but reports means and medians for the number of times each peak was fragmented in total. Across both the same beer experiment and the 6-4 beer experiment and both restrictive and permissive parameters in both tables, we can see that compared to TopN Exclusion, Non-Overlap tends towards fragmenting peaks more on average. The same can be said of Intensity Non-Overlap compared to Non-Overlap. This is congruent with the results in Figures 6 and7 which give a different view of the data in Table 9, and can again potentially be attributed to the slower decay in fragmentation events per injection seen in Tables 7 and 8.

Additionally, in the same beer case, it seems that the count of peaks in the lowest range, $5000 - 10^4$, is lower for TopN Exclusion, but the counts in the $10^5 - 10^6$ range and above are higher, which suggests these peaks have been observed at higher intensities due to the increased number of MS1 scans. However, the opposite behaviour is seen in the 6-4 beers case so this may have just been an experimental artifact or it may still hold true but cannot be observed here due to the increased complexity of this experiment. Nonetheless this may explain the anomaly observable in the $5000 - 10^4$ row of the permissive same beer segment of Table 9 where the median value for injections covered in is 0. While ordinarily we would expect the median number of injections covered in to be higher for our new methods, an increased number of zeroes raises the question of how TopN Exclusion can attain higher coverage. However, if some of the peaks that were covered were moved to a higher intensity bin and the coverage was a result of that, then this would make sense. There is no zero median in the same row of Table 10 but this may indicate, for example, that some identified peaks were fragmented below the minimum fragmentation intensity by a coincidence of the isolation window covering them. Finally, note that we can see from these tables that there is an extreme bias towards fragmenting peaks at higher intensities. This is to be expected as all three methods build on TopN's assumption that high intensity implies an interesting target for fragmentation, but additional fragmentation spectra would be more useful for peaks at lower intensities, so future method development may wish to account for this.

Based on these results we might tentatively conclude that the new methods are much more thorough

in exploring relatively "strong" peaks compared to TopN Exclusion, and thus will get higher intensity coverage on these in particular, as well as higher intensity coverage in general. In multi-sample cases (rather than just multi-injection cases with the same sample) revisiting may also confer an advantage in coverage. However, in cases where we must very rapidly obtain sample coverage on a very dense sample without caring so much about the intensity coverage, TopN Exclusion may be preferable. Trying to find the right balance of broadening coverage vs revisiting in hopes of increasing coverage/intensity coverage may guide the development of future controllers. However, while this evidence may give us some indication of the final behaviour of our controllers, it is insufficient by itself. A real example of a sample dense with interesting peaks likely has a very different distribution of intensities. Therefore in this case we might also expect Intensity Non-Overlap, etc to revisit the same targets less frequently, also broadening their coverage. Further work is needed to determine the specifics of the behaviour in this case.

Scan Counts — Same Beer						
	**TopN Exclusion**		**Non-Overlap**		**Intensity Non-Overlap**	
Run Index	MS1 Scans	MS2 Scans	MS1 Scans	MS2 Scans	MS1 Scans	MS2 Scans
1	377	6293	328	6433	328	6433
2	1277	3556	342	6418	329	6485
3	1568	2590	352	6354	335	6384
4	1681	2186	368	6205	346	6320
5	1759	1839	358	6368	354	6289
6	1820	1589	378	6256	352	6331
7	1892	1388	393	6233	360	6306
8	1984	1126	399	6158	360	6299
9	2084	879	413	6103	368	6254
10	2189	619	412	6139	361	6269
11	2250	467	415	6068	363	6268
12	2313	308	483	5845	359	6229
13	2349	218	463	5809	368	6159
14	2364	198	469	5922	365	6226
15	2384	147	451	5951	363	6188
16	2388	123	513	5717	363	6186
17	2400	108	452	5918	360	6257
18	2403	95	456	5906	365	6235
19	2404	81	530	5705	353	6255
20	2408	79	546	5513	360	6173

Scan Counts — Repeated Different Beers						
	**TopN Exclusion**		**Non-Overlap**		**Intensity Non-Overlap**	
Run Index	MS1 Scans	MS2 Scans	MS1 Scans	MS2 Scans	MS1 Scans	MS2 Scans
1	377	6293	328	6433	328	6433
2	980	4470	329	6480	328	6489
3	1384	3193	328	6467	328	6478
4	1360	3256	334	6464	329	6455
5	1594	2397	332	6375	328	6420
6	1686	2227	344	6420	331	6469
7	1653	2244	368	6348	336	6409
8	1588	2464	355	6362	338	6419
9	1905	1447	331	6396	328	6437
10	1687	2021	344	6326	330	6399
11	1891	1431	352	6244	331	6388
12	2155	806	379	6263	334	6423
13	1926	1310	375	6223	337	6418
14	1793	1789	404	6177	336	6409
15	2231	518	340	6344	329	6406
16	1919	1287	380	6053	331	6276
17	2147	752	380	6175	331	6365
18	2360	221	404	6158	340	6395
19	2151	686	401	6151	338	6336
20	1966	1306	436	6039	337	6345
21	2358	177	340	6285	331	6335
22	2127	748	390	6128	332	6262
23	2299	333	401	6084	330	6314
24	2402	104	419	6113	337	6391

Table 7: Number of scans per injection during the simulated experiment, separated by the fragmentation strategy used. Note that Non-Overlap and Intensity Non-Overlap are using the WeightedDEW variant so direct comparison can be made to the lab experiments.

Scan Counts — Same Beer						
	**TopN Exclusion**		**Non-Overlap**		**Intensity Non-Overlap**	
Run Index	MS1 Scans	MS2 Scans	MS1 Scans	MS2 Scans	MS1 Scans	MS2 Scans
1	348	6330	299	5941	298	5922
2	1043	4124	297	5863	299	5923
3	1322	3199	301	5848	298	5918
4	1450	2823	296	5866	308	5857
5	1521	2559	295	5858	297	5891
6	1054	4262	294	5833	298	5901
7	1354	3347	299	5812	307	5821
8	1627	2277	333	5641	296	5861
9	1626	2253	296	5864	294	5839
10	1659	2198	328	5692	293	5808

Scan Counts — Repeated Different Beers						
	**TopN Exclusion**		**Non-Overlap**		**Intensity Non-Overlap**	
Run Index	MS1 Scans	MS2 Scans	MS1 Scans	MS2 Scans	MS1 Scans	MS2 Scans
1	337	6350	298	5909	293	5819
2	757	5038	298	5914	298	5903
3	1221	3568	298	5918	298	5915
4	1281	3374	299	5923	300	5959
5	1456	2804	300	5945	299	5924
6	1496	2504	295	5843	294	5823
7	1447	2744	296	5881	295	5850
8	1387	2993	309	5807	299	5836
9	1539	2508	305	5851	295	5849
10	1504	2609	301	5874	297	5880
11	1599	2318	298	5892	296	5874
12	1702	1856	303	5776	293	5718
13	1586	2307	299	5834	291	5776
14	1476	2702	295	5851	291	5761
15	1607	2272	297	5861	292	5783
16	1539	2488	299	5888	293	5811
17	1645	2160	322	5776	292	5778
18	1901	1245	293	5793	284	5632
19	1623	2169	307	5767	287	5681
20	1518	2553	300	5800	287	5690
21	1645	2144	306	5737	286	5673
22	1588	2326	312	6188	289	5728
23	1702	1960	312	6183	290	5709
24	1988	960	310	6091	279	5534

Table 8: Number of scans per injection during the lab experiment, separated by the fragmentation strategy used.

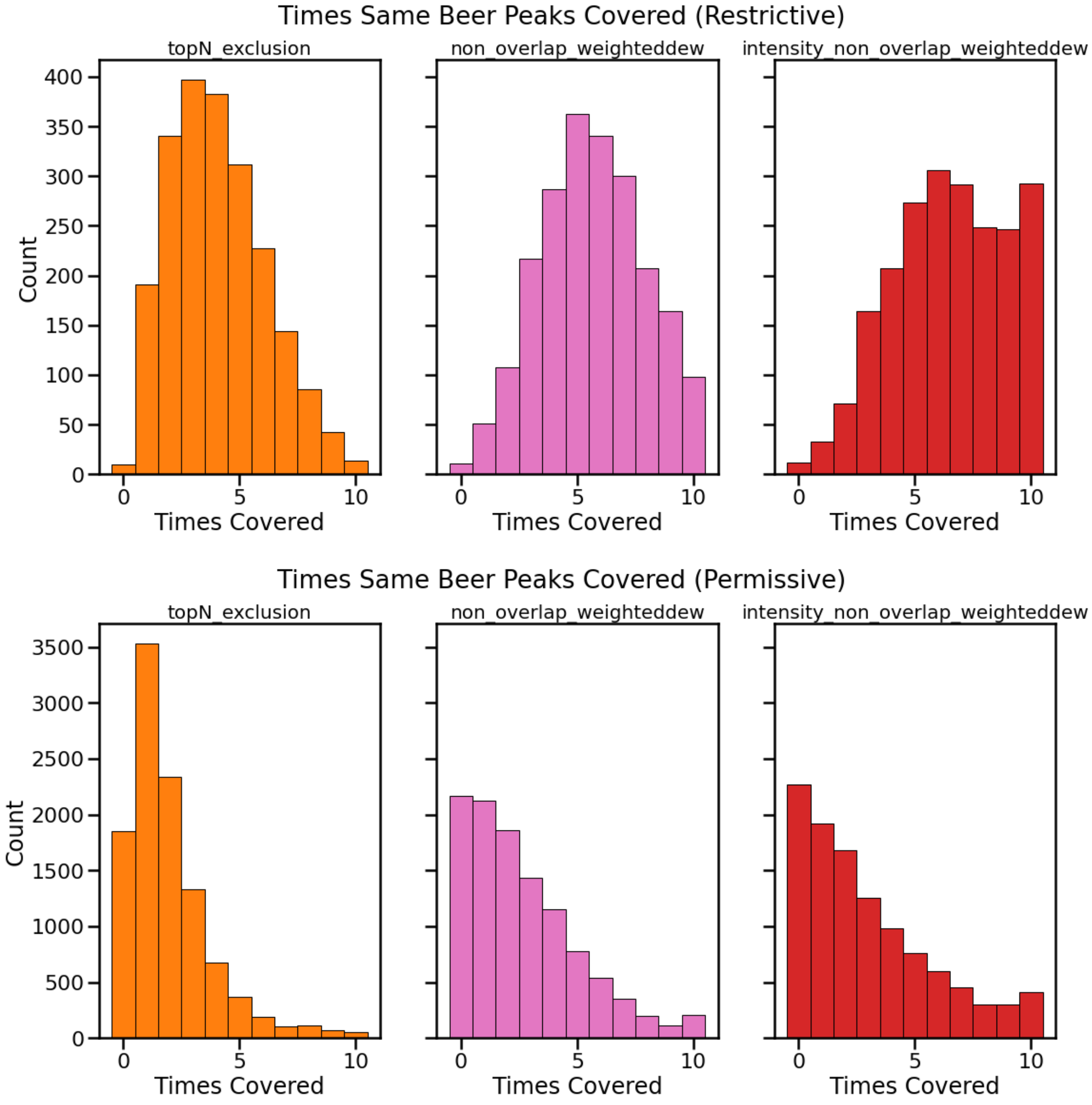

Figure 6: Counts of number of different injections each peak was covered in during the lab experiment where the same beer was repeated ten times. Top: restrictive peak-picking. Bottom: permissive peak-picking.

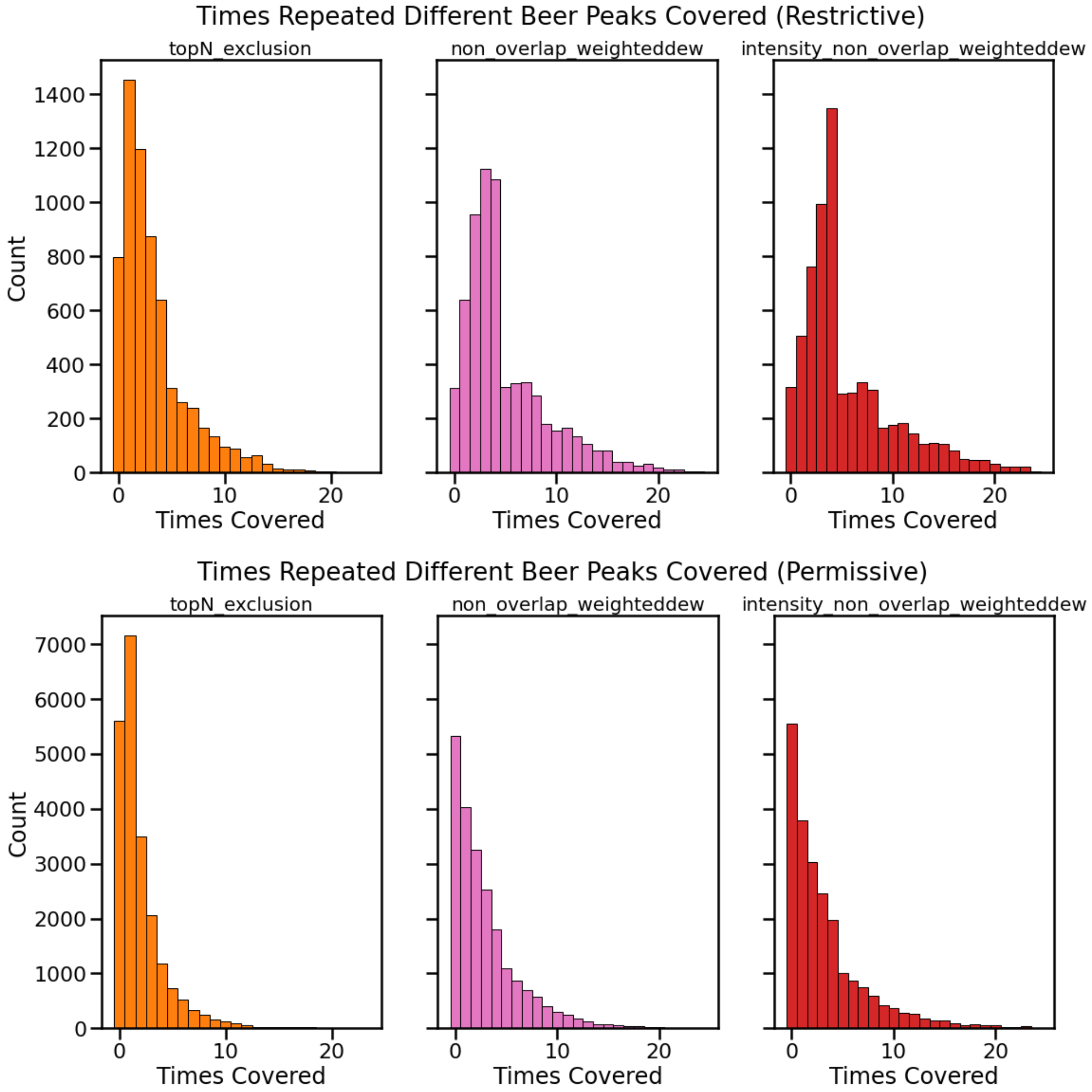

Figure 7: Counts of number of different injections each peak was covered in during the lab experiment where four beers were each repeated six times. Top: restrictive peak-picking. Bottom: permissive peak-picking.

Times Same Beer Covered by Intensity (Restrictive)									
	TopN Exclusion			Non-Overlap			Intensity Non-Overlap		
Range	Count	Mean	Median	Count	Mean	Median	Count	Mean	Median
$5000 - 10^4$	133	2.24	2.00	226	4.67	5.00	232	6.06	6.00
$10^4 - 10^5$	1219	3.89	4.00	1230	5.53	5.00	1217	6.15	6.00
$10^5 - 10^6$	633	4.42	4.00	551	5.97	6.00	557	6.87	7.00
$10^6 - 10^7$	136	5.57	6.00	120	7.12	7.00	121	8.10	8.00
$\geq 10^7$	22	7.95	8.00	16	9.69	10.00	16	9.88	10.00

Times Repeated Different Beers Covered by Intensity (Restrictive)									
	TopN Exclusion			Non-Overlap			Intensity Non-Overlap		
Range	Count	Mean	Median	Count	Mean	Median	Count	Mean	Median
$5000 - 10^4$	1087	0.91	1.00	1039	2.68	2.00	1118	3.69	3.00
$10^4 - 10^5$	3421	2.74	2.00	3338	4.23	3.00	3277	4.75	4.00
$10^5 - 10^6$	1687	4.88	4.00	1779	6.31	5.00	1777	6.93	6.00
$10^6 - 10^7$	273	8.27	8.00	309	10.94	11.00	297	11.89	12.00
$\geq 10^7$	44	12.89	13.00	47	15.96	17.00	43	17.51	19.00

Times Same Beer Covered by Intensity (Permissive)									
	TopN Exclusion			Non-Overlap			Intensity Non-Overlap		
Range	Count	Mean	Median	Count	Mean	Median	Count	Mean	Median
$5000 - 10^4$	1960	0.76	0.00	2104	2.08	1.00	2130	2.32	1.00
$10^4 - 10^5$	7382	2.03	2.00	7440	2.70	2.00	7382	2.98	2.00
$10^5 - 10^6$	1394	2.41	2.00	1229	3.07	3.00	1255	3.83	3.00
$10^6 - 10^7$	177	3.53	3.00	148	4.56	4.00	154	5.89	6.00
$\geq 10^7$	26	7.00	7.00	18	9.00	10.00	18	9.67	10.00

Times Repeated Different Beers Covered by Intensity (Permissive)									
	TopN Exclusion			Non-Overlap			Intensity Non-Overlap		
Range	Count	Mean	Median	Count	Mean	Median	Count	Mean	Median
$5000 - 10^4$	4989	0.55	0.00	4910	1.19	0.00	4509	1.69	0.00
$10^4 - 10^5$	14179	1.89	1.00	13863	2.77	2.00	14014	3.04	2.00
$10^5 - 10^6$	2980	3.73	3.00	3315	5.11	4.00	3565	5.37	4.00
$10^6 - 10^7$	329	5.20	5.00	383	6.73	6.00	387	8.24	8.00
$\geq 10^7$	49	10.55	11.00	55	12.55	12.00	51	14.76	15.00

Table 9: Table showing relationship between peak intensity and the average number of injections a peak was covered in. For each of the lab experiments and each of the peak-picking parameter sets, the table is segmented into several intensity ranges. A peak is deposited into a given range if its maximum observed intensity (not fragmentation intensity) during the run of each controller fell within that range. For each of these ranges, the table lists the count of peaks in that range and the mean/median number of injections in which any peak in that range was covered.

Times Same Beer Fragmented by Intensity (Restrictive)									
	**TopN Exclusion**			**Non-Overlap**			**Intensity Non-Overlap**		
Range	Count	Mean	Median	Count	Mean	Median	Count	Mean	Median
$5000 - 10^4$	133	5.26	5.00	226	18.06	10.00	232	19.89	12.00
$10^4 - 10^5$	1219	8.92	7.00	1230	16.47	10.00	1217	17.55	10.00
$10^5 - 10^6$	633	9.35	7.00	551	15.15	11.00	557	17.58	12.00
$10^6 - 10^7$	136	11.05	9.00	120	17.92	13.50	121	19.88	16.00
$\geq 10^7$	22	37.64	24.00	16	71.94	38.00	16	83.69	39.50

Times Repeated Different Beers Fragmented by Intensity (Restrictive)									
	**TopN Exclusion**			**Non-Overlap**			**Intensity Non-Overlap**		
Range	Count	Mean	Median	Count	Mean	Median	Count	Mean	Median
$5000 - 10^4$	1087	2.07	1.00	1039	13.03	6.00	1118	13.99	7.00
$10^4 - 10^5$	3421	6.29	4.00	3338	16.05	8.00	3277	16.66	8.00
$10^5 - 10^6$	1687	11.10	8.00	1779	18.54	12.00	1777	20.21	13.00
$10^6 - 10^7$	273	18.35	15.00	309	29.75	21.00	297	32.21	23.00
$\geq 10^7$	44	44.75	32.50	47	68.94	43.00	43	95.40	56.00

Times Same Beer Fragmented by Intensity (Permissive)									
	**TopN Exclusion**			**Non-Overlap**			**Intensity Non-Overlap**		
Range	Count	Mean	Median	Count	Mean	Median	Count	Mean	Median
$5000 - 10^4$	1960	2.12	1.00	2104	6.55	2.00	2130	8.34	2.00
$10^4 - 10^5$	7382	4.56	2.00	7440	7.06	3.00	7382	7.86	3.00
$10^5 - 10^6$	1394	5.62	2.00	1229	8.52	3.00	1255	9.94	4.00
$10^6 - 10^7$	177	6.53	4.00	148	9.86	6.00	154	12.45	8.00
$\geq 10^7$	26	28.77	14.50	18	60.83	32.00	18	70.72	32.50

Times Repeated Different Beers Fragmented by Intensity (Permissive)									
	**TopN Exclusion**			**Non-Overlap**			**Intensity Non-Overlap**		
Range	Count	Mean	Median	Count	Mean	Median	Count	Mean	Median
$5000 - 10^4$	4989	1.28	1.00	4910	4.62	1.00	4509	5.99	1.00
$10^4 - 10^5$	14179	3.96	2.00	13863	7.48	3.00	14014	8.71	3.00
$10^5 - 10^6$	2980	6.92	4.00	3315	11.24	6.00	3565	12.23	6.00
$10^6 - 10^7$	329	8.80	6.00	383	13.54	9.00	387	16.32	10.00
$\geq 10^7$	49	33.06	22.00	55	47.71	25.00	51	70.06	31.00

Table 10: Table showing relationship between peak intensity and the total number of times a peak was fragmented. For each of the lab experiments and each of the peak-picking parameter sets, the table is segmented into several intensity ranges. A peak is deposited into a given range if its maximum observed intensity (not fragmentation intensity) during the run of each controller fell within that range. For each of these ranges, the table lists the count of peaks in that range and the mean/median number of times any peak in that range was fragmented in total.

5.3 Extra Beers+Urines

Having drawn all results to this point from the same pool of data, we now ask about how performance generalises on different data. While we cannot feasibly run another large-scale lab experiment, we can re-simulate any data we have fullscans for. For this we use a previously published data set [4], which we used to test our methods prior to having any lab results to generate re-simulated data from. Although this dataset also uses beers, they are a different set from those in Table 1, and were run in a significantly different experimental context, with a different mass spectrometer (Q-Exactive) and with different instrument settings

— the details can be found in the original publication [4]. We again fixed the scan lengths of our simulations to the average scan lengths of the real TopN runs, 0.28 (MS1) and 0.13 (MS2).

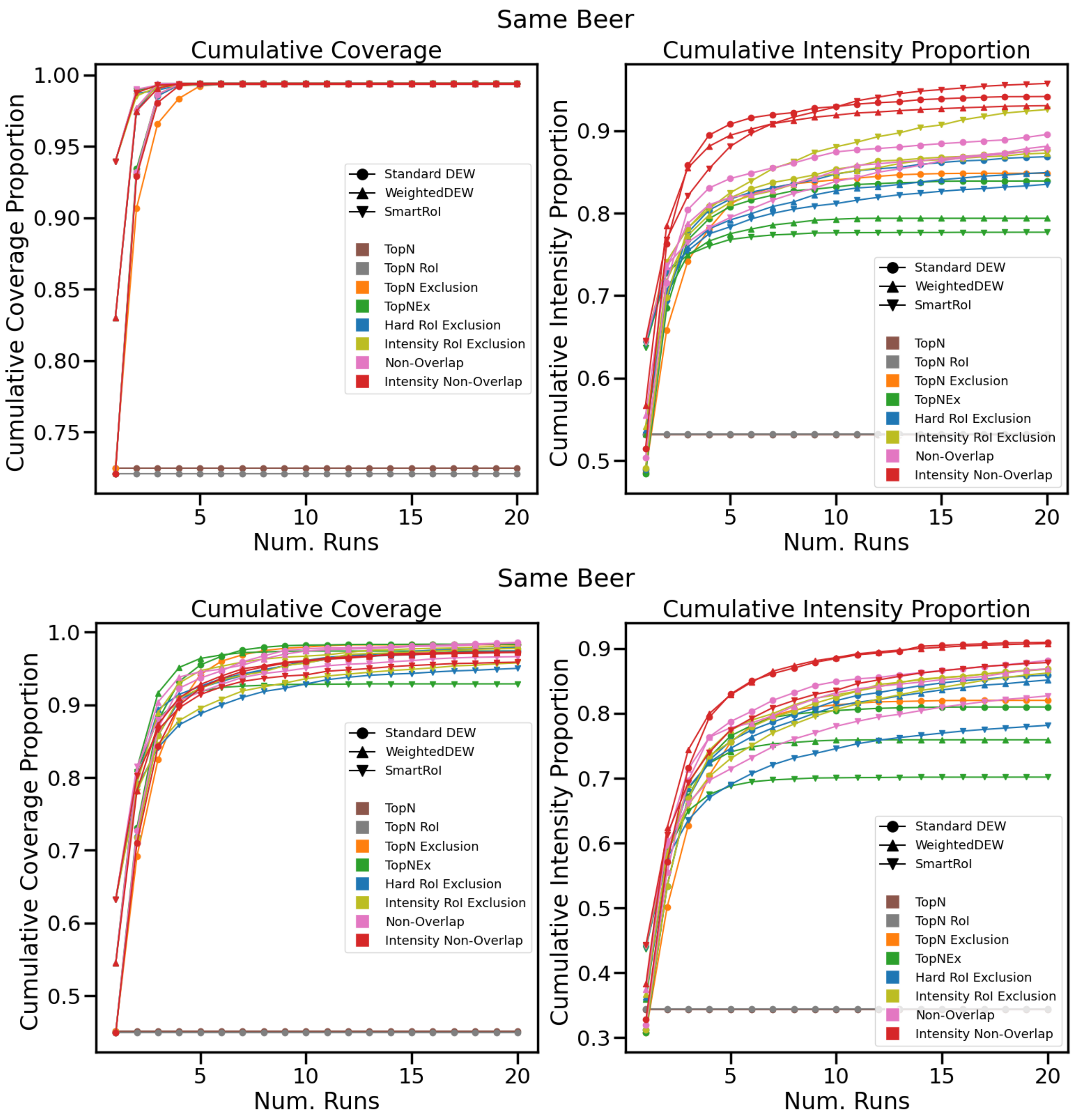

Figure 8: Simulated experiment using the alternative beers with the same beer repeated for ten injections. Top: restrictive peak-picking. Bottom: permissive peak-picking.

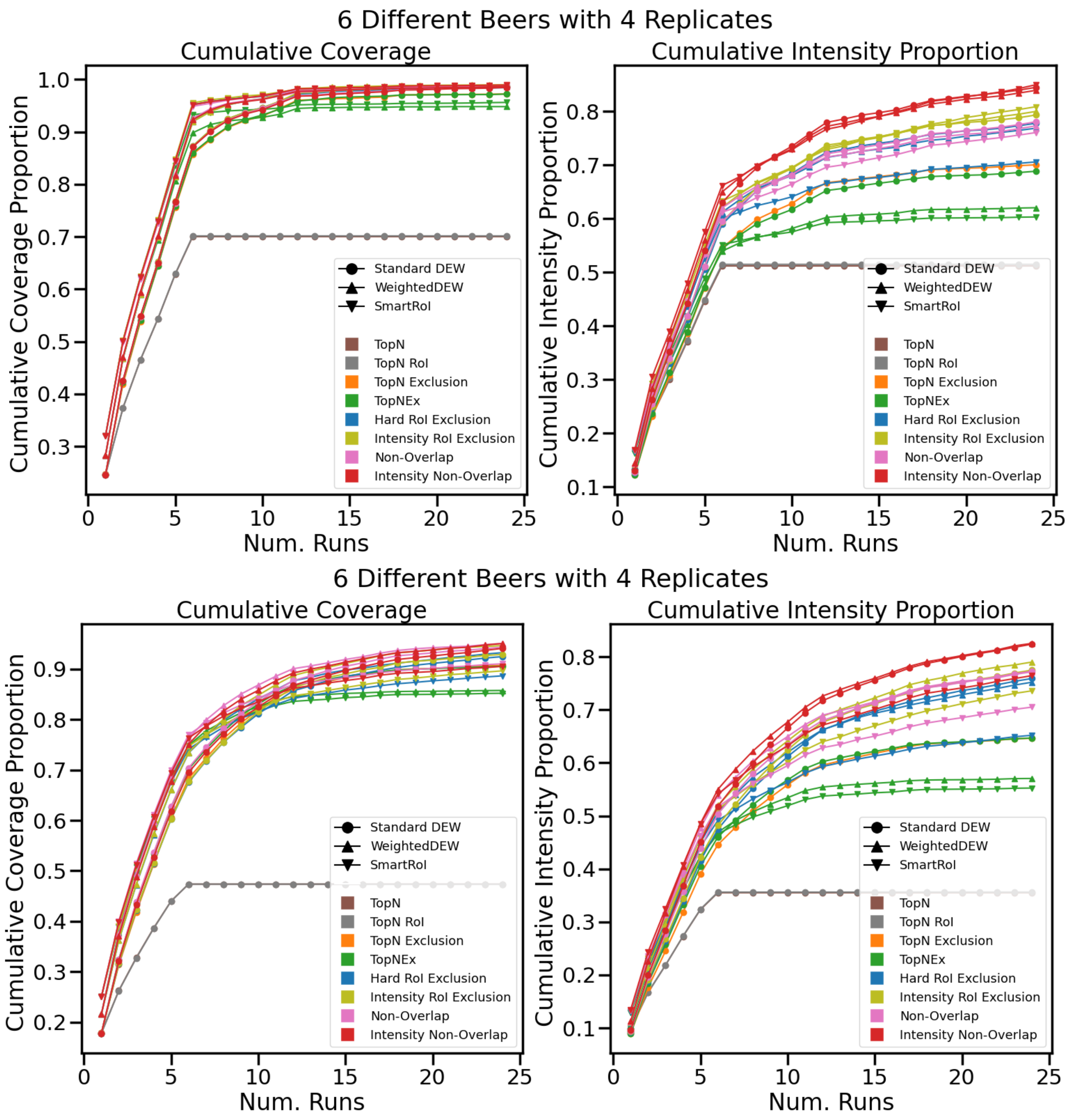

Figure 9: Simulated experiment using the alternative beers with six different beers each repeated four times. Top: restrictive peak-picking. Bottom: permissive peak-picking.

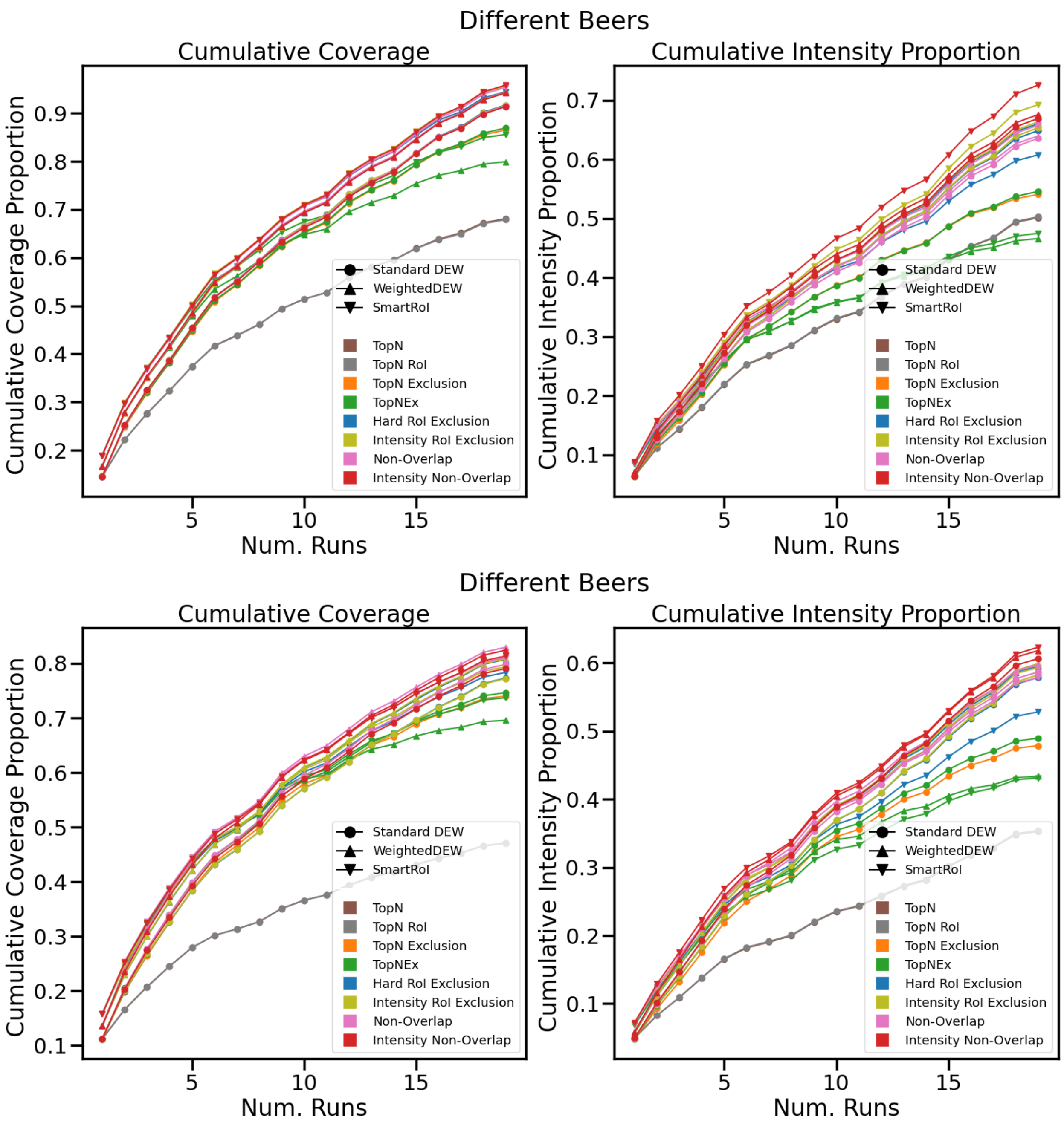

Figure 10: Simulated experiment using the alternative beers with nineteen different beers run once each. Top: restrictive peak-picking. Bottom: permissive peak-picking.

Figures 8 and 9 show results on these beers for both the same beers and 6-4 beers cases we have already explored. Figure 10 leverages the fact that this dataset contains 19 beers to perform a new kind of experiment where each beer is only run once. The equivalent with only six beers would be to read the 6-4 plot up to the sixth injection. The top plots in Figures 8, 9 and 10 show the results evaluated with the restrictive peak-picking parameters; the bottom plots show them with the permissive parameters. The same main conclusions can be observed once again. TopN only gains performance while new samples are injected, and overall performs significantly worse than multi-sample methods even prior to this point. Intensity methods easily perform best in intensity coverage, with Intensity Non-Overlap clearly dominant in all six cases. One

notable difference is that although we observed in Section 5.2 that the permissive parameter set increased the coverage of the TopN Exclusion variants relative to the new methods, the same pattern is not as pronounced here. In fact, it seems to only be clearly exhibited in the same beer case, but even in this case the Non-Overlap methods are close in performance, and in the other cases they are significantly stronger. It would therefore seem that this effect generated by the permissive parameters and the overall performance of the controllers are significantly dependent on the input data, and perhaps future work can isolate the reason why, to guide appropriate method use. Peak-peaking for the same beer case produced 1705 identified peaks with the restrictive parameters with the restrictive parameters, and 6901 with the permissive. For the 6-4 case, 5004 restrictive and 17471 permissive identified aligned peaks were produced. For the 19 different beers, 8474 restrictive and 27730 permissive identified aligned peaks were produced.

However, this dataset does not *only* contain beers. It also contains a number of human urines run in the same experimental context — now we can investigate the performance of our methods on a different kind of metabolic sample. Figures 11, 12 and 13 show the same experiments, but with urines instead of beers, and using 15 urines in the different urine case compared to 19 beers. Again, the top plots in Figures 11, 12 and 13 show the results evaluated with the restrictive peak-picking parameters; the bottom plots show them with the permissive parameters. Despite using a different type of sample these results have a very similar profile to the beer results we have just seen, and the same conclusions seem to apply, though notably the best DEW variant seems to vary more strongly between them. Overall this suggests that these results may generalise to other kinds of data and experimental setups, and that the advantages of Intensity Non-Overlap are consistent.

Peak-peaking for the same urine case produced 1142 identified peaks with the restrictive parameters with the restrictive parameters, and 5531 with the permissive. For the 6-4 case, 3903 restrictive and 15264 permissive identified aligned peaks were produced. For the 15 different urines, 7389 restrictive and 26682 permissive identified aligned peaks were produced.

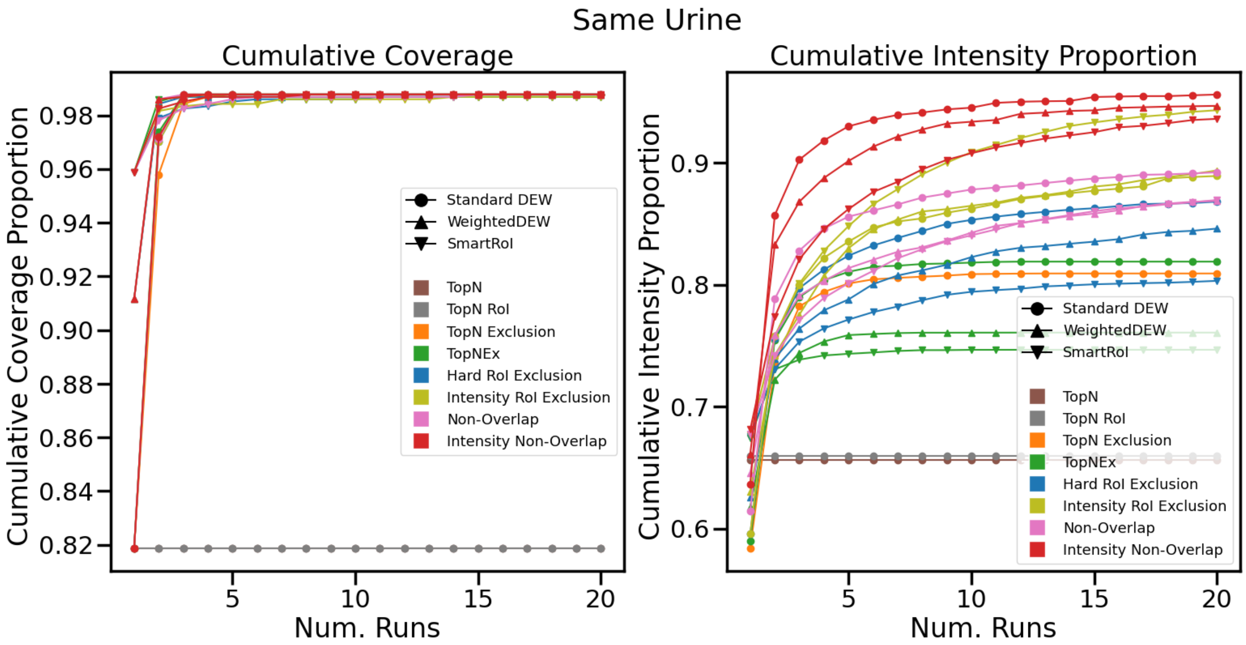

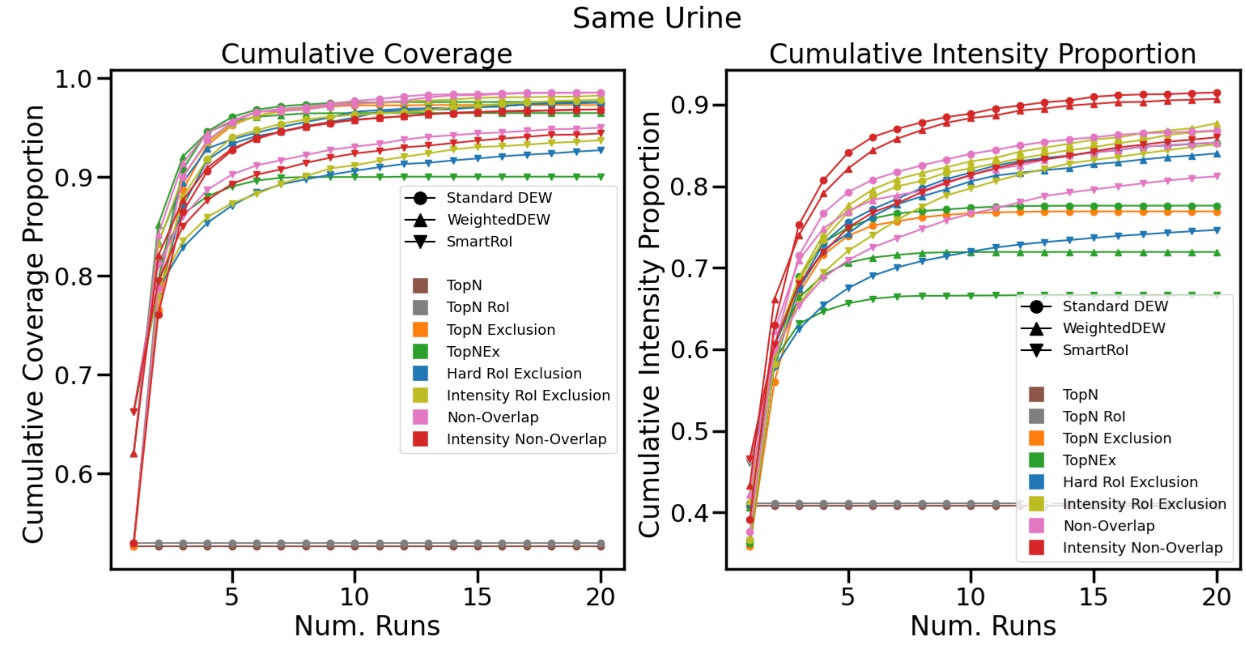

Figure 11: Simulated experiment with the same urine repeated for ten injections. Top: restrictive peak-picking. Bottom: permissive peak-picking.

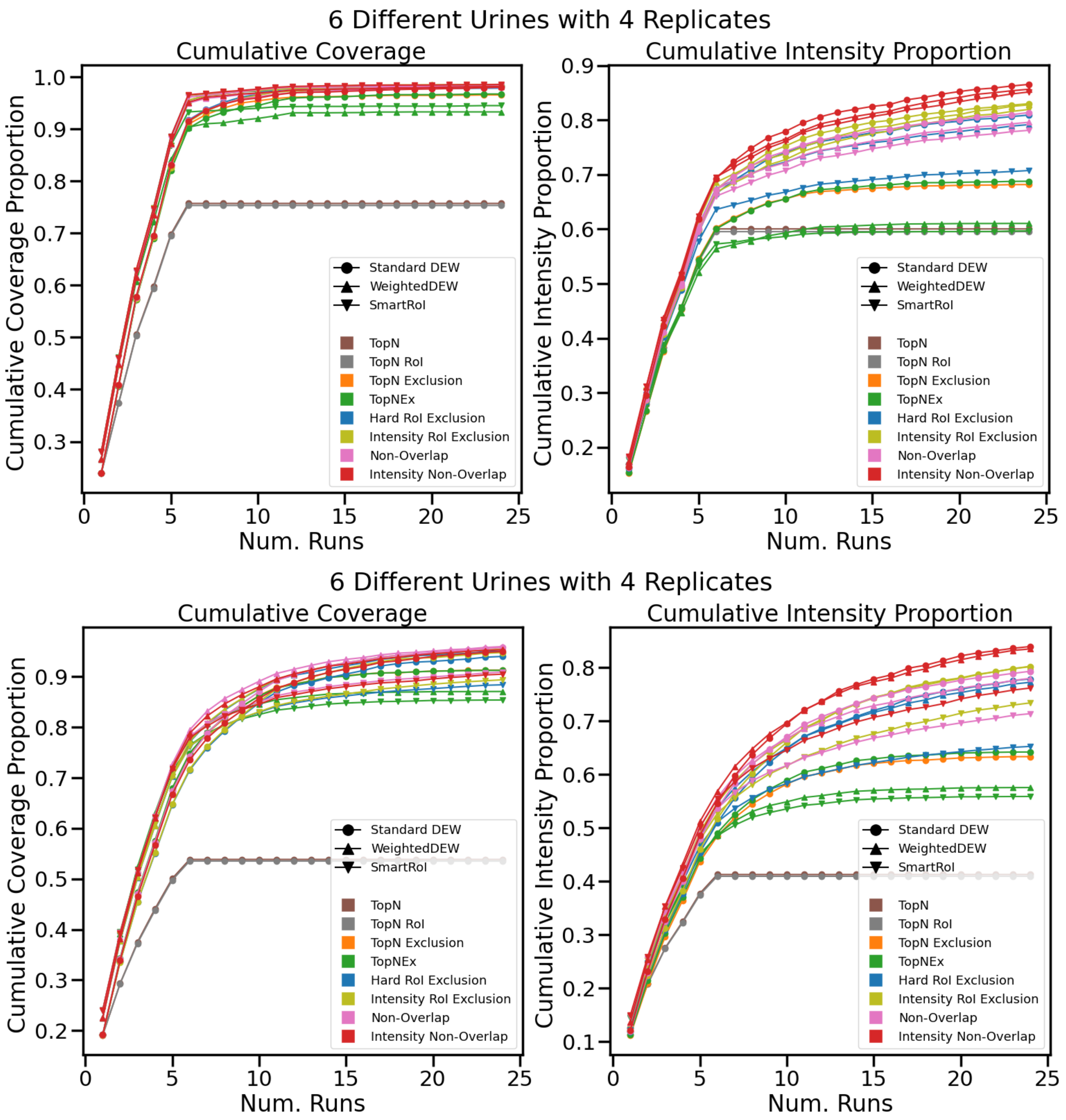

Figure 12: Simulated experiment with six different urines each repeated four times. Top: restrictive peak-picking. Bottom: permissive peak-picking.

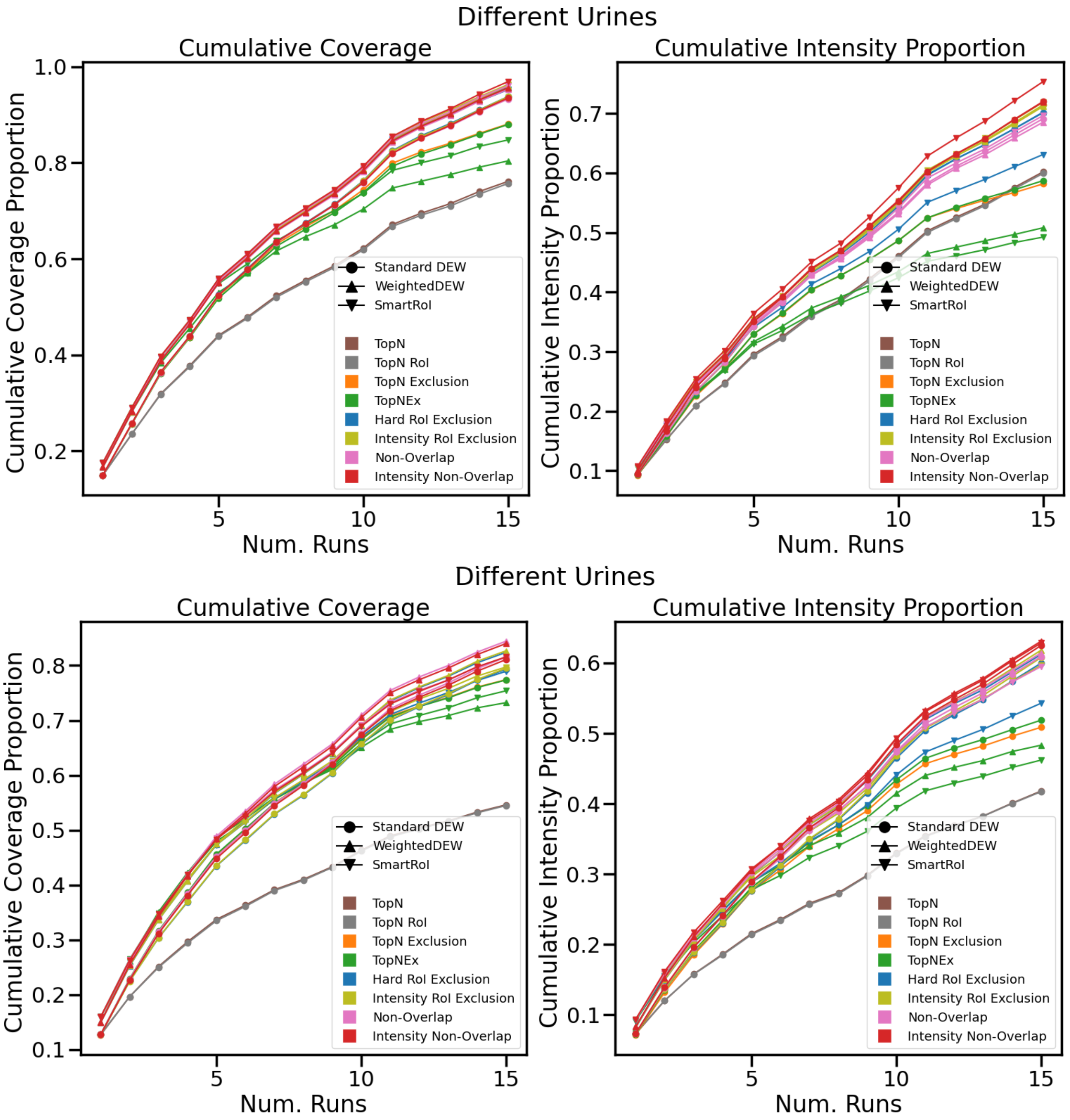

Figure 13: Simulated experiment with fifteen different urines run once each. Top: restrictive peak-picking. Bottom: permissive peak-picking.

5.4 Comparing Simulated and Real Results

Simulated results are easy to produce in large quantities, however there may be limitations to what factors they can realistically model and in this work we have used our lab results as a form of external validation against unknown sources of error. For example, the results we have presented did not model retention time drift in the simulated environment nor did we introduce other kinds of noise — when generated from a fullscan, each sample would appear exactly the same except for interpolating scans that appeared at different times as a result of the fragmentation strategy's choices. This relative predictability makes the results easier

to interpret and reproduce exactly. More generally, in a simulated environment certain properties can be turned on or off or adjusted to investigate hypotheses — in this way simulated and lab results naturally complement each other. Our results being consistent in both a pure environment where we specified only relatively limited assumptions, and in a real-world setting where many uncontrollable sources of error are potentially present, suggests there is a deeper structure to the data accurately captured by the simulation and which our methods take advantage of.

However, for direct comparison, it may still be necessary to account for differences between the environments that have a minor net effect. An example of this is the processing times. In the simulated environment, processing time is ignored and the scan length used is instead drawn from a user-specified distribution. Naturally, in the real environment, we cannot just ignore processing overhead and it is added to the MS1 scan on each cycle. As we stated in the Results section of the main manuscript, in simulation our MS1 and MS2 times were fixed to be the average times previously measured in [1], 0.59s and 0.19s respectively, because results with consistent times are easier to interpret and reproduce. This eliminates the effect of what processing overhead there would be in a real environment and thus slightly overestimates the performance of our new, more processing-heavy methods. It would in principle be possible to adjust the scan times of each method to account for this, but it is hard to know what number to use exactly given this is hardware and implementation dependent, and a full empirical study on timings to produce representative figures would be quite involved.

**Average Scan Lengths (seconds, 3 decimal places)**		
Method	Avg. MS1 Length	Avg. MS2 Length
Fullscan (1)	0.568	N/A
Fullscan (2)	0.549	N/A
Fullscan (3)	0.550	N/A
Fullscan (4)	0.549	N/A
TopN (4)	0.585	0.193
TopN Exclusion (2)	0.628	0.191
Hard RoI Exclusion WeightedDEW (4)	0.959	0.191
Intensity RoI Exclusion WeightedDEW (4)	0.979	0.190
Non-Overlap WeightedDEW (2)	0.990	0.194
Intensity Non-Overlap WeightedDEW (2)	1.086	0.194

Table 11: Average length in seconds of each scan in the lab experiments extracted from the .mzMLs. Parenthetical numbers after method indicate which batch each method was run in. Each batch produced one fullscan for each beer sample used, and the times reported here average the MS1 scan times for the first six beers for each batch (i.e. the six used in the 6-4 experiment). Non-fullscan methods were averaged over the 24 .mzMLs from the 6-4 experiment.

The results in Table 11 show the average scan times extracted from the .mzMLs produced by our lab experiments. All our new methods show a significant slowdown of around 0.4s on an approximately 0.6s MS1 scan time, and Intensity Non-Overlap by an additional 0.1s, resulting in fewer scans and likely worse overall performance. It is not surprising that Intensity Non-Overlap would cause a slowdown given it is the most processing-heavy method, though a full analysis of this slowdown would require more than comparing these averages. This is because for Intensity Non-Overlap the processing times are dependent on the number of and positions of exclusion boxes and therefore the specific sample(s) injected and the number of injections (some of these effects can be seen in Table 12). However, the 0.4s jump starting from Hard RoI Exclusion is much larger, which is quite surprising given Hard RoI Exclusion is functionally very similar to TopN Exclusion other than the inclusion of RoIs. We can eliminate it being a batching issue given that e.g. Intensity Non-Overlap and TopN Exclusion were run on a separate day from Hard RoI Exclusion and TopN. Therefore the most probable culprit is an implementation issue in the RoI-building (previously introduced in [1]) or some of the other basic scaffolding for our methods. This will likely be fixed in future versions of ViMMS but it is impossible to backport this fix to the lab results without re-running the experiments, so the results must be

interpreted with this in mind. Therefore the real results most likely slightly underestimate the performance of our new methods.

**Intensity Non-Overlap WeightedDEW Average Scan Lengths (seconds, 3 decimal places)**				
Injection Number	Beer Sample	Repeat	Avg. MS1 Length	Avg. MS2 Length
1	1	1	0.998	0.197
2	2	1	1.004	0.193
3	3	1	0.996	0.193
4	4	1	0.975	0.193
5	5	1	0.987	0.193
6	6	1	1.03	0.195
7	1	2	1.039	0.194
8	2	2	1.039	0.193
9	3	2	1.042	0.194
10	4	2	1.032	0.193
11	5	2	1.036	0.193
12	6	2	1.101	0.195
13	1	3	1.099	0.194
14	2	3	1.11	0.194
15	3	3	1.097	0.194
16	4	3	1.086	0.193
17	5	3	1.104	0.193
18	6	3	1.194	0.195
19	1	4	1.185	0.194
20	2	4	1.172	0.194
21	3	4	1.188	0.194
22	4	4	1.156	0.193
23	5	4	1.153	0.194
24	6	4	1.281	0.196

Table 12: Average length in seconds of each scan in the lab experiments extracted from the .mzMLs for Intensity Non-Overlap WeightedDEW, and with times for each injection presented separately.

Taken together, the simulated results slightly *overestimate* the performance of our new methods, and the lab results slightly *underestimate* them. Despite this the pattern we can observe between these two sets of results has not changed, so the effect size must be quite small. Consider a duty cycle of one MS1 of approximate length 0.59s and 20 MS2s of approximate length 0.19s each for a total of 4.39s. Then an increase of 0.5s, although nearly doubling the MS1 scan time, is only an increase of slightly over 10% to the length of a full duty cycle, which might explain why the negative effect to our new methods isn't more prominent. The additional slowdown to Intensity Non-Overlap in Table 12 is at most approximately 0.3s and therefore even more minor, meaning that while more precise setting of the timings in simulation may help explain minor differences, it is not necessary to understand the overall behaviour. Additionally, the value for $N = 20$ was chosen by *simulated* experiments, so our optimisation procedure in Supplementary Section 4 was not influenced by a bias to make the duty cycle longer and reduce the effect of processing time.

6 Algorithms

6.1 DEW Indicators

In WeightedDEW, a DEW is extended to be split into two adjacent time intervals with the first beginning at t_f and with lengths d_0 and d_1. In the first interval, normal DEW exclusion occurs. In the second, a $[0, 1]$-bounded weight is instead applied to the intensity, equal to the proportion of the rt-distance the precursor has from the second interval's starting-point $t_f + d_0$. Equation 1 shows $f_{ex}(p, Ex)$, which can be directly substituted for I_{ex} — note that our implementation applies this formula only to DEWs and not to multi-sample exclusion windows, which act as before.

$$f_{ex}(p, Ex) = \begin{cases} 1 & \text{if } p \text{ is not within a DEW} \\ \frac{t_p - (t_f + d_0)}{d_1} & (t_f + d_0) < t_p < (t_f + d_0 + d_1) \\ 0 & t_f < t_p \leq (t_f + d_0) \end{cases} \quad (1)$$

We found that WeightedDEW weights were sensitive to changes in their relationship with logarithms in e.g. Intensity RoI Exclusion (similarly to non-overlap, the weight worked best as a power on the unlogged modified intensity) so this motivated the placements of many of the logarithms in our equations.

SmartRoI builds on RoI-based DEWs by replacing the regular DEW with a system built on two new parameters, α and β. α controls the proportion by which a RoI's intensity must be higher than the previous fragmentation intensity λ_f: β controls the proportion of λ_{max} it must have fallen below, where λ_{max} is the highest point since the last fragmentation. If λ_r has not changed by more than either of these thresholds, r is excluded from fragmentation. Equation 2 shows $I_s(r)$ which can be substituted in the same way as $f_{ex}(p, Ex)$.

$$I_s(r) = \begin{cases} 0 & \text{if } r \text{ has been fragmented and } (\frac{\lambda_r}{\lambda_f} < \alpha) \text{ and } (\frac{\lambda_r}{\lambda_{max}} > \beta) \\ 1 & \text{otherwise} \end{cases} \quad (2)$$

6.2 Algorithmic Details

Our controllers for our new fragmentation strategies rely on some simple computational geometry. In all cases, for each active RoI in the current scan, we must find all non-DEW exclusion windows related to it, so that we can then compute a scoring function for it. TopN Exclusion, Hard RoI Exclusion and Intensity RoI Exclusion all treat the last precursor in the RoI as a point, then ask which exclusion windows it falls into. TopN Exclusion and Hard RoI Exclusion only want the boolean value of whether the precursor falls into any window, to compute the indicator function: Intensity RoI Exclusion must know all of the windows the precursor falls into so that it can retrieve the maximum of their intensities. For Non-Overlap and Intensity Non-Overlap, we must retrieve all exclusion windows overlapping a given query RoI to extract their shared area.

6.2.1 Filtering Exclusion Windows

For all controllers we must perform this search in real-time for every precursor in a given MS1 scan. An MS1 scan takes around 0.6 seconds on our instrument (see Table 11 for approximate timings) so we would ideally like processing time to be on the order of $10^{(-2)}$ or less. However, given that over the course of 20 successive injections the number of exclusion windows can reach the 100,000s, and we must search this set of windows for *every* precursor in the current scan, a naive containment check where we simply iterate through every (precursor, window) pair is not practical. Therefore we separate the space into a discrete grid of fixed-size boxes, and each of these grid-box stores which of the exclusion windows overlap it. Simple arithmetic then computes which of these grid-boxes a precursor falls into, and then we need only manually check that the precursor is contained within the small subset of exclusion windows associated with its grid-box. This data structure is updated entirely offline, between injections, when all exclusion windows for that injection are added to the total.

A simple example is to imagine dividing the space into four, then for each precursor only checking the exclusion windows overlapping the quadrant the precursor falls into. If the exclusion windows were evenly

distributed, then this would divide the number needing to be searched by four. However, when querying an entire RoI for area exclusion it may lie across multiple grid-boxes, and we must take the union of their contents before checking them for intersection. But in our simple example, even if the query RoI lay across two quadrants, then we would still only need to search half the number of exclusion windows for intersection.

By quartering the space, we have split it once on each dimension, but we extend this idea and split it more times to reduce the set of containment/intersection checks further. However, more splits is not always better. Increasing the number of grid-boxes also creates a memory overhead (and processing overhead in managing that memory) and making the grid-boxes smaller also increases the number of them that exclusion windows will be duplicated between, requiring additional processing for the query RoI to filter them down into a single set. This is only a heuristic measure with a performance increase bounded from above by some constant factor, and is sensitive to the number of splits and the distribution of exclusion windows. More splits also implies a quadratic growth in the number of grid-boxes and hence overhead. Still, we found this grid performed better in practice than using Python's *intervaltree* package once for each dimension, or the *r-tree* package. Faster implementations may be possible with alternative implementations of these data structures, segment trees or other principled methods of searching 2D space for point containment/rectangle overlap.

6.2.2 Area Calculations

After locating all exclusion windows overlapping a given query RoI, Non-Overlap and Intensity Non-Overlap must then perform area calculations on them. Intensity Non-Overlap must separate all the subregions where a unique combination of exclusion windows overlaps the query RoI — Non-Overlap requires the area where only the query RoI is present and so is a special case of this more general procedure. Although these areas themselves are not rectangular, they form a disjoint collection of rectilinear polygons (informally, shapes whose sides can all be aligned with the axes) which can be covered by non-overlapping (axis-aligned) rectangles. By doing so, we can use only rectangles to describe the areas where only a particular combination of rectangles (e.g. RoIs/exclusion windows) overlap, despite these regions having more complicated shapes. In our case this allows us to compute the area of these regions just by summing the areas of these rectangles. (An example of an alternative algorithm would be to calculate the areas of all rectangular intersecting regions without subtracting any overlapping subregions, then update them starting from the region with the most intersections and working outwards.)

It is simple to decompose two overlapping rectangles into non-overlapping rectangles: the region in which they overlap is just a rectangle, and the 0-2 non-overlapping rectilinear polygons can be covered by 0-4 rectangles. More complicated situations arise when we consider the intersection of three or more boxes and the various combinations of their overlapping and non-overlapping regions. Then an obvious algorithm is to consider the boxes one-at-a-time, maintaining a set of non-overlapping boxes after every iteration. An algorithm of this sort allows updates fully online, but in our case the set of exclusion windows is only updated at the end of every injection. During injections, we need only consider query RoIs one-at-a-time without modifying the set of split exclusion windows — we only need to split the exclusion windows in batches between injections. Additionally, ideally we would like to cover the areas using the minimum number of rectangles necessary, but this simple recursive scheme will not in general produce output that is optimal in this sense.

To fit these requirements we use a form of line-sweep algorithm. For each rectangle, there are two endpoints on each dimension where the rectangle begins and ends (i.e. each rectangle is the cartesian product of two 1D intervals). We first sort all x-endpoints and then iterate through this ordered list. As rectangles begin, we store them as "active" rectangles, and as they end, we remove them from this storage. Whenever we would update the "active" rectangles, we can then sort their y-endpoints and iterate through them similarly. If we simply emit one rectangle for each adjacent pair of y-coordinates we iterate over this way, we will end up with a set of split non-overlapping rectangles — it is not possible for necessary splits to our rectangles to appear in locations where no original rectangle had an edge. However, this will lead to obvious cases where rectangles will be split unnecessarily. For example, if two rectangles intersected on the x-dimension but not the y-dimension (i.e. a vertical line could be drawn intersecting both, but not a horizontal one), they would be partitioned into smaller rectangles whenever the other began or ended despite this being unnecessary.

Therefore we must do bounds-checking of active rectangle y-bounds, and in order to quickly query the active rectangles we store them in an interval tree. However, note that x-endpoints in this active tree will often partially overlap. When encountering a new incidence of this we can emit a box with height equal to the length of the entire interval, truncate said interval so it now only covers the non-overlapped length, and then create a new interval for the overlapping length. We therefore maintain *two* interval trees, which respectively contain the original active intervals and the split active intervals. Then when we receive a new endpoint, we can truncate all overlapping intervals in the split tree and emit a new rectangle for each, then repopulate the "missing" space using new intervals made by performing a y-sweep over the intervals returned by a query to the tree with the originals. In the geometric metaphor of the line-sweep, we can think of a vertical line sweeping along the x-endpoints. As it contacts them, a horizontal line performs a vertical sweep only along the length of the new endpoint, and after that, the vertical line "fills" the rectangle behind it. When endpoints only partially overlap, these fills split into two. By continuing this procedure to its end, we eventually fill in all the new rectangles i.e. obtain a set of non-overlapping rectangles.

6.3 Worked Example

To illustrate the concept of Non-Overlap, let us take the (not necessarily realistic) example shown in Figures 14, 15 and 16. These figures show an example where we have three boxes a (red), b (blue) and c (yellow). On the left is shown a breakdown of these three boxes into non-overlapping rectangular subregions, coloured to show their parents, and on the right is shown an intensity heatmap, where on a colourscale from grey to yellow to red each subregion is coloured according to the most intense parent box present. Figure 14 just shows this information for all boxes. Box a can be seen to be least intense, and c the most. Suppose we have previously fragmented some point in each of a and b previously (i.e. they are exclusion windows, and their intensity is their maximum fragmentation intensity) and we are interested in computing a score for whether we should fragment box c (i.e. it is an active RoI, and its intensity is the intensity of its current precursor).

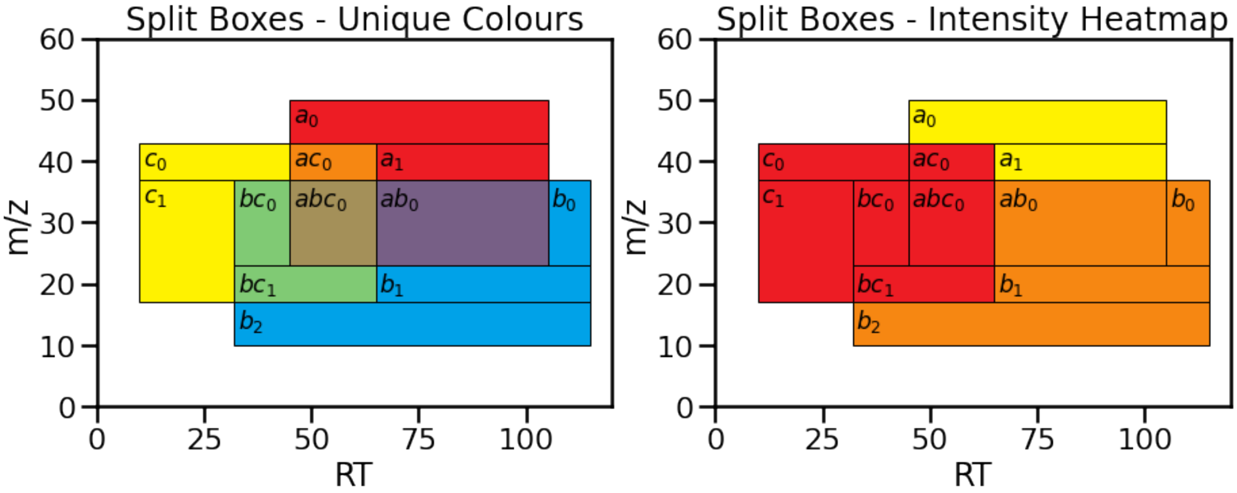

Figure 14: *Left:* A dummy example of how three overlapping boxes can be split into subregions, for illustration purposes. The three original boxes are coloured red, blue and yellow, and their shared areas interpolate their colours. *Right*: A heatmap of the intensities of the same boxes.

Then for the Non-Overlap score, we would use only the regions which are not covered by any other box (sum of all boxes c_i whose names only contain c, e.g. $c_0, c_1, ...$) as a proportion of the total area (sum of all boxes c_i^* with a name containing c, e.g. $c_0, c_1, ..., ac_0, ...bc_0, bc_1, ...abc_0, ...$). The boxes used for the numerator are highlighted in colour at their original intensity in Figure 15. Let λ_{mod} be the log modified intensity, and all other symbols as in the main manuscript. Then in this case:

$$\lambda_{mod} = log(\lambda_c) \cdot \frac{\sum_i area(c_i)}{\sum_i area(c_i^*)}$$

$$\lambda_{mod} = log(\lambda_c) \cdot \frac{area(c_0) + area(c_1)}{area(c_0) + area(c_1) + area(ac_0) + area(bc_0) + area(bc_1) + area(abc_0)}$$

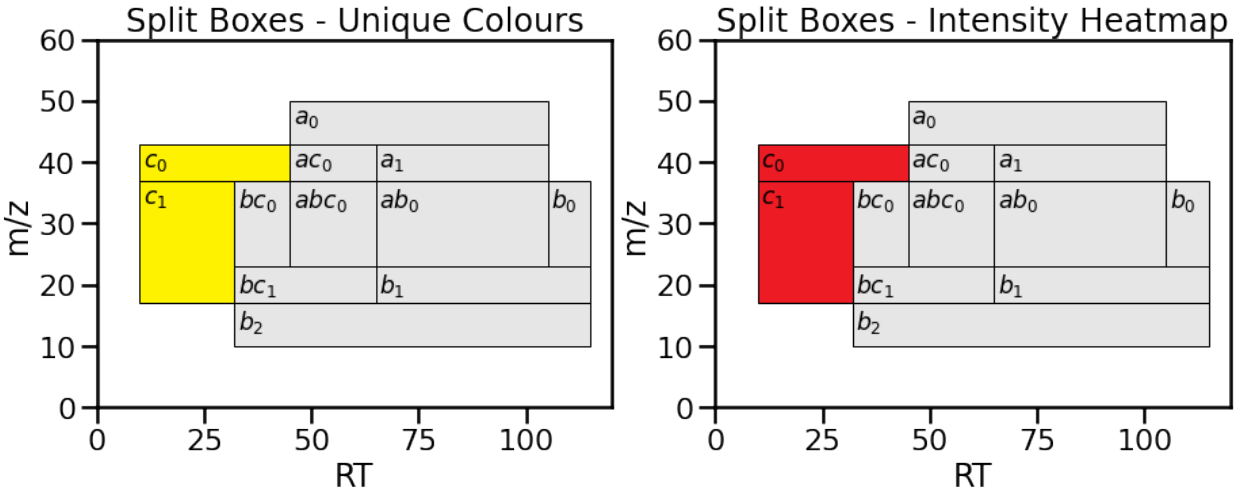

Figure 15: Example of how the Non-Overlap score would be calculated for c (the leftmost, yellow box) in Figure 14. Anything unused for the calculation of the numerator is marked in grey. Non-Overlap exclusively uses the regions where only c is present, and uses their intensities unmodified.

Having obtained the Non-Overlap modified intensity value from this, we need only apply our within-sample exclusion filters (DEW/SmartRoI/WeightedDEW) and intensity filter to get the final score. In addition to these values, Intensity Non-Overlap will also use the regions overlapped by a and b, subtracting their intensities from the regions in c (thus all boxes with c in their label will be used in both the numerator and denominator). To assign intensities to these regions, for example, λ_{cab} is the difference between the current precursor intensity of the RoI c and the maximum fragmentation intensities of exclusion windows a and b. This term may be negative (due to a or b being fragmented at a higher intensity) so we floor its contribution to the equation to zero. That the intensities of the overlapped regions are reduced can be seen in Figure 16. Now:

$$prop(c, B) = \frac{\sum_i area((cB)_i)}{\sum_i area(c_i^*)}$$

$$\lambda_{mod} = log\left(\sum_B max\left(0,\ \lambda_{cB}^{prop(c,B)}\right)\right)$$

$$\begin{aligned} exp(\lambda_{mod}) = {} & max\left(0,\ pow\left(\lambda_c, \frac{area(c_0) + area(c_1)}{area(c)}\right)\right) \\ & + max\left(0,\ pow\left(\lambda_{ca}, \frac{area(ac_0)}{area(c)}\right)\right) \\ & + max\left(0,\ pow\left(\lambda_{cb}, \frac{area(bc_0) + area(bc_1)}{area(c)}\right)\right) \\ & + max\left(0,\ pow\left(\lambda_{cab}, \frac{area(abc_0)}{area(c)}\right)\right) \end{aligned}$$

Once we apply the filters to this modified intensity we will get the final score.

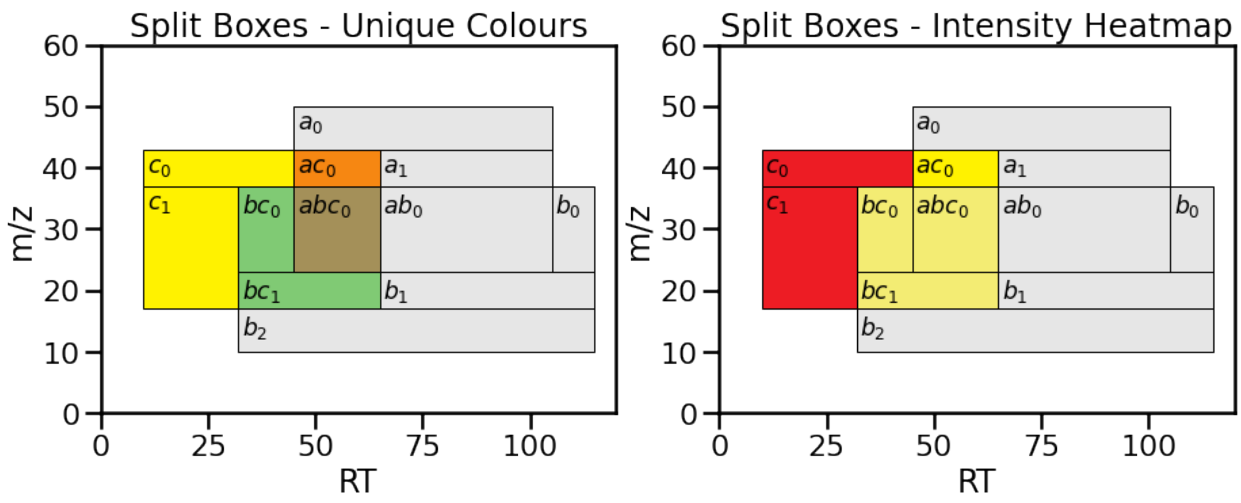

Figure 16: Example of how the Intensity Non-Overlap score would be calculated for c (the leftmost, yellow box) in Figure 14. Anything unused for the calculation of the numerator is marked in grey. Note that all of the boxes touched by c are used in the intensity area calculation. Also note that the overlapping areas used in Intensity Non-Overlap, but not Non-Overlap, are at decreased intensity compared to Figure 14 as they use the difference between c (query RoI) and a and b (exclusion windows) where they are present.

References

[1] V. Davies, J. Wandy, S. Weidt, J. J. J. van der Hooft, A. Miller, R. Daly, and S. Rogers. Rapid development of improved data-dependent acquisition strategies. *Analytical Chemistry*, 93(14):5676–5683, 2021. PMID: 33784814.

[2] O. D. Myers, S. J. Sumner, S. Li, S. Barnes, and X. Du. One step forward for reducing false positive and false negative compound identifications from mass spectrometry metabolomics data: New algorithms for constructing extracted ion chromatograms and detecting chromatographic peaks. *Analytical Chemistry*, 89(17):8696–8703, 2017. PMID: 28752754.

[3] T. Pluskal, S. Castillo, A. Villar-Briones, and M. Oresic. MZmine 2: modular framework for processing, visualizing, and analyzing mass spectrometry-based molecular profile data. *BMC Bioinformatics*, 11:395, July 2010.

[4] J. J. J. van der Hooft, J. Wandy, F. Young, S. Padmanabhan, K. Gerasimidis, K. E. V. Burgess, M. P. Barrett, and S. Rogers. Unsupervised discovery and comparison of structural families across multiple samples in untargeted metabolomics. *Analytical Chemistry*, 89(14):7569–7577, July 2017.
